# Diversity of inhibitory and excitatory parvalbumin interneuron circuits in the dorsal horn

**DOI:** 10.1097/j.pain.0000000000002422

**Published:** 2021-07-28

**Authors:** Mark A. Gradwell, Kieran A. Boyle, Tyler J. Browne, Andrew M. Bell, Jacklyn Leonardo, Fernanda S. Peralta Reyes, Allen C. Dickie, Kelly M. Smith, Robert J. Callister, Christopher V. Dayas, David I. Hughes, Brett A. Graham

**Affiliations:** a Faculty of Health, School of Biomedical Sciences & Pharmacy, University of Newcastle, Callaghan, Australia; b Hunter Medical Research Institute (HMRI), New Lambton Heights, New South Wales, Australia; c Department of Cell Biology and Neuroscience, Rutgers, The State University of New Jersey, Piscataway, NJ, United States; d W.M. Keck Center for Collaborative Neuroscience, Rutgers, The State University of New Jersey, Piscataway, NJ, United States; e Institute of Neuroscience Psychology, College of Medical, Veterinary & Life Sciences, University of Glasgow, Glasgow, United Kingdom; f Department of Neurobiology and the Pittsburgh Center for Pain Research, University of Pittsburgh, Pittsburgh, PA, United States

**Keywords:** presynaptic inhibition, postsynaptic inhibition, parvalbumin, allodynia, circuits, optogenetics, brainbow

## Abstract

Parvalbumin-expressing interneurons (PVINs) in the spinal dorsal horn are found primarily in laminae II inner and III. Inhibitory PVINs play an important role in segregating innocuous tactile input from pain-processing circuits through presynaptic inhibition of myelinated low-threshold mechanoreceptors and postsynaptic inhibition of distinct spinal circuits. By comparison, relatively little is known of the role of excitatory PVINs (ePVINs) in sensory processing. Here, we use neuroanatomical and optogenetic approaches to show that ePVINs comprise a larger proportion of the PVIN population than previously reported and that both ePVIN and inhibitory PVIN populations form synaptic connections among (and between) themselves. We find that these cells contribute to neuronal networks that influence activity within several functionally distinct circuits and that aberrant activity of ePVINs under pathological conditions is well placed to contribute to the development of mechanical hypersensitivity.

## Abstract 2

Supplemental Digital Content is Available in the Text.

Parvalbumin expressing dorsal horn interneurons in mouse can be divided into similar-sized inhibitory and excitatory subpopulations with diverse connectivity and roles in spinal sensory processing circuits.

## 1. Introduction

The dorsal horn of the spinal cord plays a key role in gating and modulating sensory input originating from primary afferents before it is relayed to supraspinal sites for perception. The principal neuronal populations in this region can be differentiated into interneurons that process and modulate sensory input at a local (spinal) level and projection neurons (PNs) that relay this information to higher centres for sensory perception. Interneurons can be further subdivided into 2 groups based on their principal neurotransmitter content: excitatory interneurons use glutamate, whereas inhibitory interneurons use GABA and/or glycine.^76^ Both populations of interneurons are highly heterogeneous in their morphology, physiological properties, neurochemistry, and genetic profiles. Recent advances in molecular genetic profiling of dorsal horn interneurons^1,37,67^ coupled with the increased use of transgenic animals have enabled researchers to target and manipulate discrete neuronal populations with great precision,^33^ leading to a better understanding of the functional role of various cell types.^1,56,60^

One population of spinal interneurons that have been widely studied are those that express the calcium-binding protein parvalbumin (PV). In the dorsal horn(DH), these cells are found primarily in lamina II inner (IIi) and III, with similar patterns of expression being described in various species including rat,^4,12,81^ cat,^3^ and mouse.^3^ Immunohistochemical studies in the rat have shown that ∼75% of PV-immunolabelled cells in laminae I-III express both GABA and glycine,^5,44^ with the remaining cells considered to be excitatory (glutamatergic) interneurons. More recent approaches using transgenic mouse lines have estimated that 70% of genetically labelled parvalbumin-expressing interneurons (PVINs) are inhibitory interneurons, with the remainder being excitatory.^1^ Inhibitory PVINs (iPVINs) are known to be important in setting mechanical thresholds under both normal and pathological conditions.^8,38,63^ Under normal conditions, these cells mediate both presynaptic (axoaxonic) inhibition of myelinated low-threshold mechanoreceptive afferents and postsynaptic inhibition of both vertical cells^8^ and PKCγ-expressing interneurons.^63^ After nerve injury, the intrinsic excitability of iPVINs is reduced and the resulting disinhibition opens circuits through which low-threshold mechanoreceptive afferent input can be relayed to lamina I. These studies demonstrate the importance of inhibition mediated by iPVINs under normal and pathological conditions, but the role of excitatory PVINs (ePVINs) in spinal circuits has yet to be determined. To address this, the aims of our study were to reassess the incidence of both ePVIN and iPVIN populations, characterise the synaptic connectivity of these cells, and define the dorsal horn circuits in which these populations influence the relay of sensory information to supraspinal sites.

## 2. Methods

### 2.1. Animals

All procedures were approved by the Animal Care and Ethics Committee at the University of Newcastle. All experimental procedures performed at the University of Glasgow were conducted in accordance with the European Community directive 86/609/EEC and UK Animals (Scientific Procedures) Act 1986. All experiments were conducted on wild-type animals (C57Bl/6), PV^Cre^ knock-in mouse line (JAX Stock #08069), or offspring of PV^Cre^ mice crossed with the Cre-dependent ChR2-YFP mouse line Ai32 (JAX Stock #012569) or the Cre-dependent tdTomato (tdTOM) reporter line Ai9 (JAX Stock #012569).

### 2.2. Immunocytochemistry in PV^Cre^;Ai9 mice

Three PV^Cre^;Ai9 mice (both sexes, 18-21 g) were perfused transcardially with fixative containing 4% formaldehyde in 0.1 M phosphate buffer. Transverse sections (60 µm thick) of the lumbar enlargement (L3-L5) were cut on a vibrating microtome and immersed for 30 minutes in 50% alcohol to enhance antibody penetration. Sections were incubated for 3 days at 4°C with primary antibodies (Supplementary Table 1, available at http://links.lww.com/PAIN/B443), washed in double-salt phosphate-buffered saline (PBS), and then incubated for a further 24 hours at 4°C with species-specific secondary antibodies raised in donkey. Sections were then washed again with double-salt PBS and mounted on glass coverslips in Vectashield antifade mounting medium (Vector Laboratories, Burlingame, CA). Primary and secondary antibodies were diluted in PBS containing 0.3% Triton X-100 and 5% normal donkey serum.

Tiled confocal stacks encompassing the entire section thickness of one dorsal horn were taken on 3 sections per animal on a Zeiss LSM 710 microscope system, using a 40× oil-immersion lens (NA = 1.3) and a z-step of 1 µm. Each channel of the resulting images (containing PV, Pax2, or mCherry immunoreactivity) was viewed separately, and all cell bodies in laminae IIi-III that were immunopositive within each channel were marked throughout the section thickness using Neurolucida software (MBF Bioscience, Williston, VT). Cells were only included if the maximal profile of their soma lay within the section thickness. Once each channel had been assessed independently, the marked cells were combined and co-expression of PV, Pax2, and/or mCherry was determined.

### 2.3. Fluorescent in situ hybridization in wild-type mice

Three wild-type C57BL/6 mice (both sexes, 18-20 g) were used for in situ hybridization experiments, as described previously.^36^ Animals were decapitated under deep isoflurane anaesthesia before their spinal cords were removed and then rapidly frozen on dry ice. Fresh frozen lumbar spinal cord segments were embedded in OCT mounting medium and then cut into 12-μm-thick transverse sections on a cryostat (Leica CM1860; Leica, Milton Keynes, United Kingdom) and mounted on Superfrost Plus slides (48311‐703; VWR, Lutterworth, United Kingdom). Multiple‐labelling fluorescent in situ hybridization was performed with RNAscope probes and fluorescent multiplex reagent kit 320850 (ACD BioTechne, Newark, CA). Reactions were performed according to the manufacturer's recommended protocol. Probes used in this study were GAD1 (Cat no. 400951), Slc17a6 (Cat no. 319171), PValb (Cat no. 421931), and CCK (Cat no. 402271). Sections were reacted with the probe combinations GAD1, Slc17a6, and PValb or CCK, Slc17a6, and PValb, revealed with Atto 550, Alexa 647, and Alexa 488, respectively. Sections were mounted in ProLong Glass antifade medium with NucBlue (Hoechst 33342; ThermoFisher Scientific, Paisley, United Kingdom). RNAscope positive and negative control probes were tested on other sections simultaneously.

Three sections per animal were selected before viewing in situ hybridization fluorescence (to avoid bias) and imaged using the ×40 oil-immersion lens with the confocal aperture set to 1 Airy unit. In each case, tile scanning of a single optical plane through the middle of the section was used to include the whole of laminae I-III. Semiautomated analysis of transcript numbers per nucleus was conducted using the cell detection and subcellular object features on QuPath software.^36^ Cell analysis was conducted only in laminae IIi and III, where a band of dense PV transcripts was present. Recognition and segmentation of individual nuclei was performed based on NucBlue staining. An additional 2 μm perimeter was added to each nucleus to allow detection of perinuclear transcripts. This additional perimeter was omitted where cells were directly adjacent to each other. Any areas with poor nuclear segmentation were excluded manually from the analysis after examination of each segmented section. Single RNA transcripts for each target gene appeared as individual puncta, and detection thresholds were adjusted manually until the markup accurately reflected the transcript distribution. Data output consisted of manual inspection of the section to ensure accuracy, followed by export of a table containing each cell's transcript numbers. This was further analysed in Microsoft Excel. Cells were defined as positive for expression of a given gene if they contained greater than 4 transcripts. Cells were classified as excitatory or inhibitory depending on expression of Slc17a6 or GAD1, respectively.

### 2.4. Intraspinal injection of Brainbow-encoding adeno-associated viruses

To visualise the somatodendritic arbors of individual PV-expressing spinal interneurons, we performed co-injections of 2 adeno-associated viruses (AAVs) carrying Cre-dependent Brainbow expression cassettes in PV^Cre^ mice (n = 5; both sexes; 18-23 g at surgery): AAV.Brainbow1 codes for eYFP and TagBFP, whereas AAV.Brainbow2 codes for mTFP and mCherry.^10^ By adopting this approach, individual cells displayed a unique colour profile based on the stochastic expression of farnesylated fluorescent proteins encoded by the AAVs.

To perform these intraspinal AAV injections, animals were anaesthetised with isoflurane (5% induction and 1.5%-2% maintenance) and placed in a stereotaxic frame. Bilateral injections were made into the L3 dorsal horns using a glass micropipette attached to a 10-µL Hamilton syringe. Injections were made through the T12-13 intervertebral space, 400 µm lateral to the midline, and 300 µm below the pial surface. For each injection, 500 nL of virus was infused at a rate of 30 to 40 nL/min using a syringe pump (Harvard Apparatus, MA). Given that the aim of this experiment was to reconstruct the morphology of individual PV-expressing cells, we used moderate titres of viruses (3.77 × 10^8^ GC for AAV.Brainbow1 and 3.72 × 10^8^ GC for AAV.Brainbow2) to achieve sparse labelling of PV neurons. By capturing only a small proportion of all PV neurons and ensuring that individual cells are labelled with a unique colour, this approach allows the morphology of individual neurons can be traced with accuracy and great fidelity.. Once injections were complete, wounds were closed, and animals were allowed to recover with appropriate analgesic administration (0.3 mg/kg buprenorphine and 5 mg/kg carprofen). All animals made uneventful recoveries from the intraspinal injection surgery.

### 2.5. Morphological analyses of Brainbow-labelled parvalbumin interneurons

Sagittal sections (60 µm thick) of the L3 spinal segment were processed for immunocytochemistry as detailed above. For the analysis of neuronal morphology, sections were incubated in a cocktail of primary antibodies to reveal 3 (TagBFP, mTFP, and mCherry) of the 4 fluorescent proteins as well as Pax2 expression to label inhibitory interneurons.^27,45^ Tiled confocal scans of Brainbow-labelled cells within laminae IIi-III were made through the full thickness of the sections using the 40× objective (1.5× zoom and 0.5 µm z-step). Cells were selected for reconstruction if (1) they demonstrated relatively strong staining for at least one fluorescent protein (to allow them to be readily distinguished from neighbouring labelled cells based on colour) and (2) their soma was found near the middle of the section in the z-axis (to ensure maximal representation of their dendritic arbor in the mediolateral plane). The presence or absence of Pax2 immunolabelling in these cells was then determined. Based on these selection criteria, the somatodendritic morphology of 30 inhibitory (Pax2-expressing) and 34 excitatory (Pax2-lacking) Brainbow-labelled PV interneurons was reconstructed in 3 dimensions using Neurolucida software (Table 1). To compare the morphology of inhibitory and excitatory PV interneurons objectively, 50 morphological parameters (5 for the soma and 45 for the dendritic arbor) were extracted from the Neurolucida reconstructions^21,39^ using Neurolucida Explorer (MBF Bioscience, Williston, VT). K-means nonhierarchical clustering was performed in Orange software (University of Ljubljana) based on these 50 morphological parameters, with the number of clusters set to 2 and cluster seeds chosen using the K-means++ algorithm. The 3D graph of the morphological variables within the K-means–derived clusters was produced in TeraPlot (Kylebank Software Ltd., Ayr, United Kingdom).

**Table 1:** Distribution of excitatory and inhibitory parvalbumin interneurons within K-means–derived clusters.

	Cluster 1	Cluster 2	Total
Excitatory	33	1	34
Inhibitory	7	23	30
Total	40	24	64

Contingency table showing the count of excitatory and inhibitory PVINs within each of the 2 K-means–derived clusters (cluster 1 and cluster 2).

To determine whether excitatory and inhibitory PV interneurons form homotypic and/or heterotypic synaptic circuits between themselves, we used antibodies to reveal 2 of the Brainbow fluorescent proteins (mTFP and mCherry) combined with immunolabelling for gephyrin and Homer1 to label inhibitory and excitatory synapses, respectively. Given that we were limited in the number of fluorophores we were able to stain for in our experiment (total of 4), we stained Pax2 immunoreactivity in the same channel as that for Homer1 because the 2 proteins can be easily differentiated based on their subcellular localisation (ie, Pax2 is expressed in the nucleus, whereas Homer1 produces punctate membrane labelling).

### 2.6. Electrophysiology

All electrophysiological studies were performed on PV^Cre^;Ai32 mice (both sexes; age = 30 ± 3 weeks). Mice were deeply anesthetized with ketamine (100 mg/kg, i.p) and decapitated, and the lumbar enlargement of the spinal cord was rapidly removed and glued to the stage of a vibrating microtome (Leica VT-1000S, Heidelberg, Germany). Sagittal or transverse slices (200 µm thick) were prepared in carbogenated ice-cold sucrose-substituted ACSF containing (in mM) 250 sucrose, 25 NaHCO_3_, 10 glucose, 2.5 KCl, 1 NaH_2_PO_4_, 1 MgCl_2_, and 2.5 CaCl_2_. All slices were incubated for 1 hour at 22 to 24°C in an interface chamber containing carbogenated ACSF (118 mM NaCl substituted for sucrose) before recordings.

Slices were transferred to a recording chamber and continually superfused (bath volume 0.4 mL; exchange rate 4-6 bath volumes/min.) with ACSF bubbled with carbogen (95% O_2_ and 5% CO_2_) to achieve a pH of 7.3 to 7.4. Recordings were obtained at room temperature (21-24°C), and neurons were visualized with Nikon FN-PT with near-infrared differential interference contrast optics connected to a camera (Jenoptik ProgRes MFcool). Recordings were made in 2 locations: (1) within the clearly discernible YFP-expressing plexus of PVINs (laminae IIi-III) and (2) superficial to the PVIN plexus (laminae I-IIo). Furthermore, 3 neuron types were targeted in recordings: (1) PVINs identified by YFP expression and ChR2-mediated photocurrents, (2) unidentified dorsal horn neurons that lacked YFP and did not exhibit photocurrents, and (3) virally labelled PNs. Slices were illuminated using a CoolLED pE excitation system that allowed visualization of YFP fluorescence, ChR2 photostimulation using a FITC filter set, and visualization of mCherry-expressing PNs with TRITC filters.

Recordings were acquired in voltage-clamp (holding potential −70 mV) or current clamp (maintained at −60 mV) using small (<20 pA) bias current injection. Patch pipettes (4-8 MΩ) were filled with a cesium chloride–based internal solution containing (in mM) 130 CsCl, 10 HEPES, 10 EGTA, 1 MgCl_2_, 2 ATP, and 0.3 GTP (pH adjusted to 7.35 with 1 M CsOH) for assessing inhibitory synaptic transmission. A potassium gluconate–based internal solution containing (in mM) 135 C6H11KO7, 6 NaCl, 2 MgCl2, 10 HEPES, 0.1 EGTA, 2 MgATP, and 0.3 NaGTP, pH 7.3 (with KOH) was used to record AP discharge or excitatory synaptic transmission (internal solution osmolarity adjusted to 280 and 300 mOsm, respectively). In some voltage-clamp recordings of photostimulation-evoked responses in PV-ChR2/YFP neurons, QX-314 bromide (5 mM) was added to the internal solution to block fast-activating voltage-gated sodium channels. This avoided ChR2-mediated unclamped spikes in the recorded PVIN cell, without blocking photostimulation-evoked discharge in neighboring PVINs. Alternatively, QX-314 was excluded when assessing action potential discharge in the current-clamp mode. Neurobiotin (0.2%) was included in both internal solutions for post hoc confirmation of neuronal morphology (Vector Laboratories, Burlingame, CA).

Data were amplified using a MultiClamp 700B amplifier (Molecular Devices, Sunnyvale, CA), digitized online (sampled at 10-20 kHz and filtered at 5-10 kHz) through an ITC-18 computer interface (Instrutech, Long Island, NY), acquired, and stored using AxoGraph X software (AxoGraph X, Sydney). After obtaining the whole-cell recording configuration, series resistance, input resistance, and membrane capacitance were calculated based on the response to a 5 mV hyperpolarising voltage step (10 ms duration) from a holding potential of −70 mV. These values were monitored at the beginning and end of each recording session, and data were rejected if values changed by more than 30%.

Photostimulation intensity was suprathreshold (16 mW) with a duration of 1 ms (controlled by transistor–transistor logic pulses), unless otherwise stated. This ensured generation of action potential discharge in PV-ChR2/YFP neurons and allowed confident assessment of postsynaptic currents in recorded neurons. To isolate monosynaptic connectivity, 1 μM TTX and 200 μM 4-AP were bath-applied to block action potential discharge and accentuate light-evoked neurotransmitter release from ChR2-expressing terminals.^17,64^ To assess the impact of PVIN activation on action potential discharge responses in unidentified neurons (ie, cells lacking YFP), a series of 3 depolarizing step currents were repeated (1000 ms or 50 ms duration, 20 pA increments, delivered every 10 seconds). During this protocol, PVINs were activated by photostimulation (16 mW, 10 ms) at the onset of second series of depolarizing steps and AP discharge was compared with the preceding and subsequent responses.

To identify lamina I PNs, a subset of animals (n = 8) underwent surgery to inject a viral tracer, specifically AAV9.CB7.CI.mCherry (viral titre = 2.5 × 1013 vg/mL), into the parabrachial nucleus (PBN) for subsequent targeted patch-clamp recording experiments. In brief, mice were anaesthetised (isoflurane, 5% induction, 1.5%-2% maintenance) and secured in a stereotaxic frame (Harvard Apparatus, Massachusetts). Two small craniotomies were performed (5.25 mm posterior to bregma, ± 1.2 mm of midline, and 3.8 mm deep from skull surface^59^), and up to 700 nL of virus was injected into each PBN using a picospritzer (PV820, WPI, Florida). Pipettes were left in place for a further 7 to 10 minutes to avoid drawing the virus sample along the pipette track. Animals recovered for 2 to 4 weeks to allow retrograde labelling of PNs. Spinal cord slices were obtained using methods described above (Electrophysiology section), and mCherry-positive neurons were visually targeted for recording and PVIN photostimulation was performed as above. The brainstem of animals was also removed and sectioned to confirm the injection site. In all cases, the injection site was focussed on the PBN.

### 2.7. Optogenetic stimulation for Fos and pERK activation mapping

The postsynaptic circuits targeted by PVINs were assessed by delivering spinal photostimulation to anaesthetised PV^Cre^;Ai32 animals and then processing spinal cords for Fos protein as described previously.^71^. Animals (n = 6) were anaesthetised with isoflurane (5% initial, 1.5%-2% maintenance) and secured in a stereotaxic frame. A longitudinal incision was made over the T10-L1 vertebrae, and a laminectomy was performed on the T13 vertebra. Unilateral photostimulation (10 mW, 10 ms pulses at 10 Hz for 10 minutes) was then delivered to the exposed spinal cord by positioning an optic fiber probe (400 nm core, 1-mm fiber length, Thor Labs, New Jersey) above the spinal cord surface using the stereotaxic frame. Photostimulation was delivered by a high-intensity LED light source attached to the probe through a patch cord. Animals were maintained under anaesthesia for either 5 minutes from the onset of photostimulation (for pERK mapping; n = 3) or 2 hours (for Fos mapping; n = 3) before transcardial perfusion with saline followed by 4% depolymerised formaldehyde in 0.1 M phosphate buffer. Sections were processed for immunocytochemistry by incubating in a cocktail of primary antibodies including chicken anti-GFP and goat anti-Fos or mouse anti-MAPK (Erk1/2). Primary antibody labelling was detected using species-specific secondary antibodies conjugated to rhodamine and Alexa 488 (Jackson ImmunoResearch, West Grove, PA). Confocal image stacks of these sections were analysed using Neurolucida for Confocal software to assess the laminar distribution of pERK-immunolabelled cells. The lamina I/IIo border was taken to be 20 µm below the dorsal white matter,^39^ and the boundary between lamina IIo and IIi was delineated using the dorsal extent of YPF immunolabelling in PVIN processes.^38^ Lamina IIi was defined as the most superficial region of the YFP plexus and deemed to have the same dorsoventral extent as lamina IIo. The dorsoventral extent of lamina III was taken as being equal to that of laminae IIi and IIo combined.

### 2.8. Electrophysiology data analysis

All data were analysed offline using AxoGraph X software (AxoGraph X, Sydney). AP discharge was classified according to previously published criteria.^31,32^ The criterion for inclusion of a neuron for analysis was an RMP more negative than −50 mV and a series resistance < 30 MΩ (filtered at 5 KHz). In the analysis of AP discharge, individual APs elicited by step-current injection were captured using a derivative threshold method (dV/dt > 15 V/second) with the inflection point during spike initiation defined as AP threshold. Rheobase current was defined as the smallest current step that elicited at least one AP, and AP latency was measured as the time difference between the stimulus onset (current injection or photostimulation) and AP threshold.

Most data assessed optically evoked excitatory postsynaptic currents (oEPSCs) and inhibitory postsynaptic currents (oIPSCs) in recorded neurons during PVIN photostimulation. When this analysis was undertaken in targeted PVIN recordings, an immediate photocurrent was also directly evoked during photostimulation followed by synaptic responses. In these instances, the baseline current was set to zero at the onset of the synaptic response, with the amplitude of photostimulation-evoked synaptic input then measured from this level. In all other recordings (ie, cells lacking ChR2/YFP), synaptic responses were measured from baseline just before photostimulation. The peak amplitude of responses was calculated from the average of 10 successive trials. A number of parameters were considered for determining whether a photostimulation-evoked synaptic input was monosynaptic or polysynaptic. The latency of oPSCs was measured as the time from photostimulation to the onset of the evoked current. The “jitter” in latency was measured as the standard deviation in latency of 10 successive trials. Importantly, the latency of monosynaptic inputs was much shorter, there was minimal jitter in the onset of responses between trials, and reliability (percentage of photostimulation trials to evoke a response) was higher than those deemed polysynaptic inputs. To assess the contribution of different neurotransmitter systems to photostimulation responses, various synaptic blockers were sequentially applied. Changes in photostimulation-evoked postsynaptic current amplitude were measured to calculate either an oIPSC_index_ or oEPSC_index_.^26^ These were calculated using the amplitude of the oPSC in the presence of the drug identified and amplitude of oPSCs before the application of that specific drug. An oPSC_index_ of 1 indicates the drug has no effect and 0 when the drug completely blocks the oPSC. Note, an oPSC_index_ of 0 was not possible for cells lacking YFP due to small variations in baseline noise; however, photocurrent zeroing before photostimulation-evoked oPSCs in PVIN recordings resulted in a reliable oPSC_index_ of 0 under drug block conditions.

### 2.9. Statistics

Statistical analysis was performed using SPSS v10 (SPSS Inc., Chicago, IL) and Prism 7 (GraphPad Software Inc., San Diego, CA). Student *t*-tests and Student–Newman–Keuls ANOVAs were used to compare variables. Data that failed tests for normal distribution and homogeneity of variance were compared using the nonparametric Kruskal–Wallis or Mann–Whitney tests. Statistical significance was set at *P* < 0.05. All values are presented as means ± SEM unless otherwise stated.

## 3. Results

### 3.1. Molecular genetic profiling of parvalbumin neurons in laminae IIi and III

**Figure 1.**
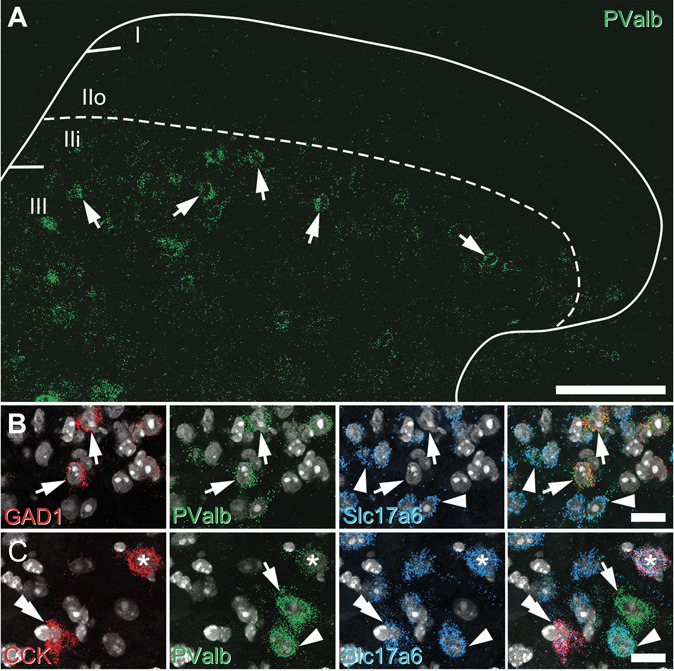
Neurochemical characterisation of parvalbumin cells in the spinal dorsal horn using fluorescent in situ hybridisation. (A) Lumbar spinal cord sections processed for fluorescent in situ hybridisation to map parvalbumin expression (PValb; green) showed most cells were concentrated in laminae IIi and III (arrows). (B) Multiple labelling with probes for GAD1 (red; inhibitory interneurons), PValb (green), Slc17a6 (blue; for excitatory interneurons), and NucBlue (gray) was used to show that approximately half of the PVINs in laminae I-III were inhibitory interneurons (arrows), with the remainder being excitatory interneurons (arrowheads). (C) Similar studies using probes to CCK (red), PValb (green), Slc17a6 (blue), and NucBlue (gray) to show that approximately 75% of excitatory PVINs co-express CCK (asterisk), but these account for only ∼25% of CCK population (double arrowhead). This field also shows an ePVIN (arrowhead) and iPVIN (arrow), neither of which co-express CCK. All images are generated from a single optical section. Scale bars (µm): A = 100; B and C = 25. iPVIN, inhibitory parvalbumin-expressing interneuron; PVIN, parvalbumin-expressing interneuron.

We have previously shown that the distribution of PV-expressing neurons in the mouse spinal dorsal horn is similar to that found in other species with most cells found in laminae IIi and III,^38^ but note that the incidence of these cells in the mouse dorsal horn is higher than that in either rat or cat.^26^ Despite this difference, immunohistochemical approaches in the rat^44^ and molecular genetic studies in the mouse^44^ estimate that 70% to 75% of PVINs are inhibitory interneurons, with the remainder being excitatory interneurons. Here, we aimed to directly resolve the relative proportions of excitatory and inhibitory interneurons within the PV population using a multiple-labelling fluorescent in situ hybridization approach (Fig. 1). Parvalbumin-expressing cells were identified using a probe for PValb, excitatory interneurons using a probe for Slc17a6 (to identify VGLUT2-expressing cells), and inhibitory interneurons using a probe for GAD1 (to identify GABAergic cells). The distribution of cells labelled with the PValb probe matched that reported previously using immunohistochemistry,^38^ with most cells being found in laminae IIi and III (Fig. 1A). For 515 PV-expressing cells analysed in sections from 3 mice (147, 153, and 215), we found that 52.7% (SEM ± 4.7%) expressed Slc17a6 (274/515: 64, 90, and 120), with the remainder expressing GAD1 (241/515; 83, 63, and 95; Fig. 1B).

Given recent molecular genetic profiling studies of mouse dorsal horn interneurons have shown that glutamatergic populations enriched in PV expression (Glut1 and Glut2) express high levels of the neuropeptide CCK,^37^ we also assessed the degree of co-expression of CCK in the ePVIN populations using in situ hybridization (Fig. 1C). Of the 201 Slc17a6-expressing PV cells analysed from 3 mice (75, 54, and 72), 74.5% (±2.3%) expressed CCK (150; 59, 40, and 51). Conversely, from a total of 556 Slc17a6 cells that express CCK in these laminae, only 27.0% express PV (59, 40, and 51). From these studies, we conclude that ePVINs account for a far higher proportion of PVINs in mouse than previously reported, and although most of these (∼75%) express CCK, ePVINs only account for approximately a quarter of CCK-expressing cells in this region.

In a complementary approach, we also assessed the offspring from a PV^Cre^ mouse line crossed with a Cre-dependent reporter line (Ai9), used to target and record from PVINs in the spinal dorsal horn.^8^ The fidelity of tdTOM expression in PV-expressing cells from these PV^Cre^;Ai9 mice was analysed in the spinal cord (dorsal horn laminae II and III and in the ventral horn), the cerebellum, and both the dentate gyrus and CA1 subfield in the hippocampus (Supplementary Fig. S1, available at http://links.lww.com/PAIN/B443). Overall, most tdTOM-expressing cells co-expressed immunolabelling for PV (94.5%, 1504 of the 1591 cells), and most PV-immunoreactive (PV-IR) cells also expressed tdTOM (68.2%; 1504 of the 2204 cells). A high proportion of PV-IR cells in the hippocampus (83.1%; ±0.7) and ventral horn of the spinal cord (86.3%; ±7.9) expressed tdTOM, and nearly all tdTOM-labelled cells in these regions were immunolabelled for PV (94.0%; ±0.9 and 97.7%; ± 0.7, hippocampus and ventral horn, respectively). By contrast, far fewer PV-IR cells in dorsal horn laminae II and III expressed tdTOM (37%; ±10.1), although most of these tdTOM cells were immunolabelled for PV (90.4%; ±2.2). We therefore find that Cre-mediated recombination in this PV^Cre^ line captures PV-expressing cells with high fidelity but that the overall proportion of PV-IR cells labelled with tdTOM is lower in the dorsal horn than other regions of the central nervous system.

Given that we have used this mouse line to record from and label iPVINs, as well as to manipulate their function using optogenetic and targeted silencing approaches,^8^ we determined whether tdTOM expression in these dorsal laminae captured iPVINs preferentially by comparing the expression pattern of a developmental marker for inhibitory interneurons, Pax2 as previously.^70^ We found that 48.0% (±2.1) of PV-IR cells in laminae II and III showed immunolabelling for Pax2, whereas 40.2% (±1.4) of cells that co-express both tdTOM and PV-IR were also immunolabelled for Pax2. These immunohistochemical findings mirror our in situ hybridization analysis above and show that there is no preferential expression of PV^Cre^-mediated reporter molecules in iPVIN and ePVIN populations of laminae II and III. We conclude that this line provides a reliable, unbiased means of studying PVINs. The resultant expression profile provides a faithful means to resolve detailed neuroanatomical and functional connectivity patterns within the complex and heterogeneous neuropil of the spinal dorsal horn.

### 3.2. Morphological features and interconnectivity of parvalbumin-expressing interneurons in laminae IIi and III

**Figure 2.**
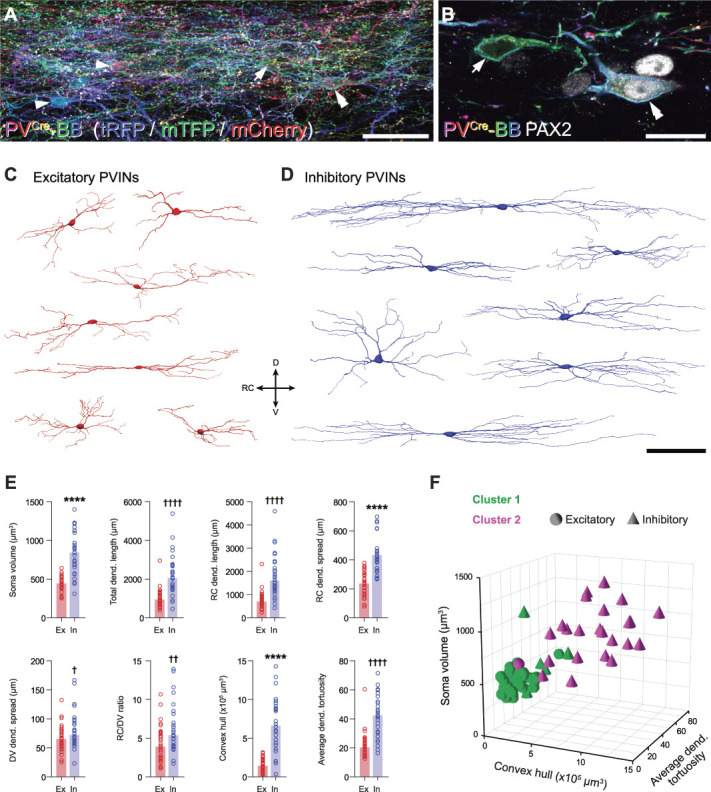
Morphometric analyses of Brainbow-labelled excitatory and inhibitory PV interneurons. (A) Example of Brainbow labelling in laminae IIi/III of a sagittal section from a PV^Cre^ mouse injected with AAV.Brainbow1 and AAV.Brainbow2, showing a dense plexus of Brainbow-labelled PV cells in laminae IIi and III. Examples of individual cell bodies within this plexus are highlighted with arrowheads. The cells highlighted with an arrow and a double arrowhead are shown at higher magnification in (B). This image is a maximum projection of 107 optical sections at 0.5 µm z-spacing. (B) Higher magnification of 2 cells outlined in panel (A) (arrow and double arrowhead), generated from a single optical section. This figure demonstrates the use of immunostaining for Pax2 to identify inhibitory (double arrowhead; Pax2-expressing) and excitatory (arrow; lacking Pax2) Brainbow-labelled PVINs. (C and D) Representative 3D reconstructions of the somatodendritic morphology of excitatory (C, red) and inhibitory (D, blue) Brainbow-labelled PVINs. DV, dorsoventral axis; RC, rostrocaudal axis. (E) Grouped scatterplots of selected morphometric parameters of all reconstructed excitatory (Ex; red; n = 34) and inhibitory (In; blue; n = 30) PVINs. Key to y-axes: Dend, dendritic; RC dend. length, total length of dendrite projecting in the rostrocaudal axis; RC dend. spread and DV dend. spread are the distances between the most distal points in the rostrocaudal and dorsoventral axes, respectively; RC/DV ratio, RC dend. spread/DV dend. spread. ****P < 0.0001 by the unpaired *t* test for normally distributed data; †*P* < 0.05, ††*P* < 0.01, ††††*P* < 0.0001 by the Mann–Whitney test for nonnormally distributed data. Bars for normally distributed data show mean, and bars for nonnormally distributed data show median. (F) Scatterplot of soma volume (y-axis) vs convex hull volume (x-axis) vs average dendritic tortuosity for all reconstructed PV interneurons (z-axis), grouped by K-means–derived cluster (cluster 1 or cluster 2; green or magenta, respectively) and neurotransmitter phenotype (excitatory or inhibitory; spheres or cones, respectively). Note that these 3 axis variables were selected for this visualisation because they are assumed to be independent of each other. Scale bars (µm): A = 50; B = 20; C and D = 100. AAV, adeno-associated virus; PV, parvalbumin; PVIN, parvalbumin-expressing interneuron.

Although some previous work has assessed PVIN morphology in filled and reconstructed or immunolabelled cells,^8,38^ these approaches can be technically demanding, rarely yield full reconstructions, and have not differentiated excitatory and inhibitory subpopulations. We therefore utilise Brainbow labelling of PVINs here to systematically compare the morphology of ePVINs and iPVINs utilising a Brainbow-labelling approach.^10^ Intraspinal injection of Cre-dependent Brainbow viral vectors labelled individual PVINs with unique colour profiles based on the stochastic expression of farnesylated fluorescent proteins encoded by the respective viruses, whereas both the cellular and subcellular localisation of various target molecules remained unaltered (Fig. 2A). We assessed the morphological features of individual Brainbow-labelled ePVINs and iPVINs from confocal image stacks, as differentiated by expression (or absence) of the inhibitory cell marker Pax2 (Fig. 2B). The somatodendritic arborisation of 64 Brainbow-labelled neurons in laminae IIi-III was traced in 3 dimensions using Neurolucida for Confocal software (MBF Bioscience, VT). Thirty of these cells expressed Pax2 and were classified as iPVINs, with the remainder classified as ePVINs. Although some overlap in the morphological features was evident between the 2 populations, iPVINs generally had larger cell bodies and dendritic arbors that extended further in the rostrocaudal (RC) axis and branched more often, compared with ePVINs (Figs. 2C–E). The soma volumes of iPVINs were significantly greater than those of ePVINs (841.8 μm^3^ vs 444.3 μm^3^, respectively, *P* < 0.0001), as were their total dendritic lengths (2054 μm vs 943 μm, *P* < 0.0001). The difference in dendritic length between these 2 populations was most apparent when measured in the RC axis (1603 µm vs 696 µm for iPVINs and ePVINs, respectively, *P* < 0.0001), with iPVINs also showing greater dendritic spread in the RC axis (433.4 µm vs 236.2 µm, *P* < 0.0001). The volume of tissue encapsulated by the dendritic arbors of iPVINs was also significantly larger than that of ePVINs (66,0654 μm^3^-144,623 μm^3^, respectively, *P* < 0.0001), whereas fractal dimension (1.12 vs 1.08, *P* = 0.0139) and tortuosity of dendrites within these volumes (42.3 vs 20.7, *P* < 0.0001) were also significantly higher for iPVINs than those of ePVINs.

To determine whether morphological features can be used to differentiate both PVIN subpopulations, we performed K-means multivariate cluster analysis on 50 morphological parameters extracted from the Neurolucida reconstructions of these cells. These comprised 5 parameters for the soma and 45 for the dendritic arbors (including those plotted in Fig. 2E; refer to Table 1 for details). By setting the number of clusters to 2, the distribution of both PVIN subpopulations within these K-means–derived clusters could be compared in an unbiased manner (Fig. 2F and Table 1). Using this approach, 97% (33/34) of ePVINs were contained within cluster 1 and 77% (23/30) of iPVINs were within cluster 2. From this, we conclude that ePVINs and iPVINs are morphologically distinct and that soma size and extent of dendritic arbors in the RC axis are two of the strongest discriminators between these populations.

**Figure 3.**
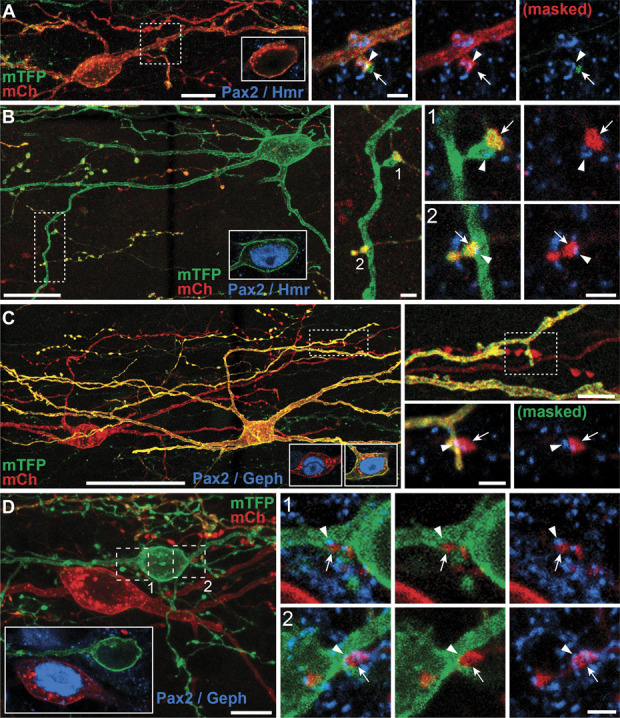
Homotypic and heterotypic synaptic connectivity between PVINs in laminae IIi and III. (A and B) Examples of homotypic synaptic connections made by ePVINs onto other ePVINs (A) and heterotypic synaptic connections onto iPVINs (B). Insets in (A and B) show the presence or absence of Pax2 (blue) in the soma of the target neuron. High-power insets of areas outlined on the target neurons show axon terminals (arrows) forming excitatory synaptic inputs on to the dendrites of excitatory (A) and inhibitory PVINs (B), respectively. Excitatory synapses are verified by the presence of immunolabelling for Homer1 (blue; arrowheads). (C and D) Examples of homotypic synaptic connections made by iPVINs onto other iPVINs (C) and heterotypic synaptic connections onto ePVINs (D). Insets in (C and D) show the presence or absence of Pax2 (blue) in soma of the presynaptic and postsynaptic neurons illustrated. High-power insets of areas outlined on the target neurons show axon terminals (arrows) forming inhibitory synaptic inputs onto the dendrites of inhibitory (C) and excitatory PVINs (D). Inhibitory synapses are verified by the presence of immunolabelling for gephyrin (blue; arrowheads). Lower-power panels are maximum projections of 35, 115, 132, and 47 optical sections for figures (A, B, C, and D), respectively, with a z-separation of 0.5 µm (A and C) or 0.3 µm (B and D). Insets detailing Pax2 immunolabelling in cell bodies are single optical sections. High-power panels detailing synaptic contacts are maximum projections generated from 3 optical sections at 0.3 µm z-steps. Scale bars (µm): A = 10 and 2; B = 20, 2 and 2; C = 50, 5 and 2; D = 10 and 2. ePVIN, excitatory parvalbumin-expressing interneuron; iPVIN, Inhibitory parvalbumin-expressing interneuron; PVIN, parvalbumin-expressing interneuron.

Having prepared this tissue to optimise structural preservation and the retention of tissue antigenicity, we also aimed to determine whether these PVIN subpopulations formed synaptic connections to each other. Immunolabelling for Pax2 was again used to differentiate iPVINs from ePVINs, and once this had been established, we were able to trace axons from these cells to determine whether they formed homotypic and/or heterotypic synaptic connections onto other Brainbow-labelled PVINs. Sparce labelling afforded in the PV^Cre^ line made it possible to trace individual axons with greater precision than would otherwise be possible. Excitatory and inhibitory synapses were visualised using immunolabelling for Homer-1 and gephyrin, respectively. Using these approaches, we found anatomical evidence for excitatory synaptic inputs derived from ePVINs onto both iPVINs and ePVINs (Figs. 3A and B, respectively) and also of inhibitory synaptic inputs from iPVINs onto both iPVINs and ePVINs (Figs. 3C and D, respectively). We conclude that the synaptic targets of both ePVINs and iPVINs include other PVINs in laminae IIi and III and that these cells form both homotypic and heterotypic connections.

### 3.3. Parvalbumin-expressing interneuron photostimulation

**Figure 4.**
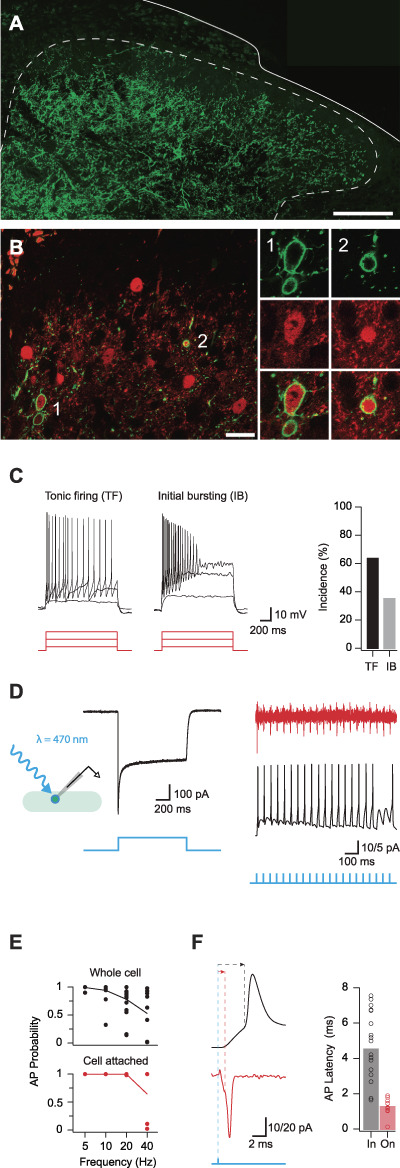
ChR2 expression and activation in PVINs. (A) Representative image showing the distribution of ChR2:YFP expression (green) in the lumbar dorsal horn of a PV^Cre^;Ai32 mouse. (B) Left panel compares ChR2:YFP expression (green) and PV-IR profiles (red). Most ChR2:YFP neurons express PV-IR (98%) examples noted (1, 2), although relatively few PV-IR profiles express ChR2:YFP (9%) (double arrow). Right images show neurons labelled “1” and “2” (from left image) at high magnification: ChR2:YFP (upper), PV-IR (middle), and merge (lower). (C) Upper traces show action potential discharge in PVINs recorded during depolarising step current injections (lower, 20, 60, and 100 pA steps shown). PVIN discharge patterns could be reliably classified as either tonic firing or initial bursting. Bar plot (right) shows incidence of PVIN discharge patterns. (D) Trace shows example photocurrent recording from a PVIN. Recorded in voltage clamp, PVINs exhibit large inward photocurrents in response to photostimulation. Inset schematic shows recording arrangement with photostimulation. (E) Traces show 1 ms photostimulation at 20 Hz reliably evokes AP discharge in PVINs in cell-attached voltage-clamp recordings (upper, red) and whole-cell current-clamp recording (lower, black). Blue trace (bottom) indicates the photostimulation protocol for each representative trace. Plots (right) compare the reliability of evoked AP discharge across a range of photostimulation frequencies using whole-cell (upper) and cell-attached (lower) recording configurations. (F) Traces compare photostimulation-evoked action potential spiking using whole-cell (upper) and cell-attached (lower) recording configurations. Note, recruitment latency (time between photostimulation and AP threshold) is shorter in the cell-attached mode. Bar graphs (right) show variability in recruitment latency using whole-cell (black) and cell-attached (red) recording configurations. Short latencies observed in the cell-attached configuration suggest that the ChR2-expressing PVINs require at least ∼2 ms to generate an AP during photostimulation. Scale bar in A = 200 μm. PVIN, parvalbumin-expressing interneuron.

To study the function of PVIN-mediated synaptic connections, we bred PV^Cre^;Ai32 mice to undertake channelrhodopsin2 (ChR2)-assisted circuit mapping. We first assessed the expression of YFP (corresponding to ChR2 expression) immunolabelling in the spinal dorsal horn of these animals. The distribution of YFP immunolabelling mirrored that of PV-IR, with a dense plexus of labelling in laminae IIi and III, but largely absent in more dorsal lamina (Fig. 4A). Most YFP/ChR2^+^ neurons were also immunolabelled for PV (98%, 133/136 and 106/109 in 2 animals, Fig. 4B). However, consistent with sparse dorsal horn expression in the PV^Cre^ mouse line, only a subset of PV-IR neurons expressed ChR2 (∼10%, 133/1420 and 106/1110 in 2 animals). Therefore, while this mouse line only captures a proportion of the entire PV population, it reliably and selectively expresses ChR2 in these cells. As highlighted above for neuroanatomical work, relatively sparse ChR2 expression among PVINs permitted detailed optogenetic analysis of connectivity within the dorsal horn. This restricted expression patterns allow photostimulation responses from individual neurons to be studied with greater precision, avoiding widespread network activity with a higher yield of cells.

Targeted recordings used a potassium gluconate–based internal solution to assess action potential discharge in ChR2/YFP-expressing PVINs. During depolarising current step injections (Fig. 4C), these neurons exhibited either tonic firing (TF) (n = 34/53, persistent AP discharge throughout the depolarizing step) or initial bursting (IB) responses (n = 19/53, AP discharge limited to depolarizing step onset). These discharge patterns match our previous work in PVeGFP mice and PV^Cre^;Ai9 mice^1,8,38^ and suggest that the ChR2/YFP-expressing cells captured by the PV^Cre^;Ai32 line are representative of the PVIN population. Photostimulation of recorded PVINs produced immediate large inward currents (photocurrents) in voltage clamp (holding potential −70 mV), and these photocurrents were sufficient to evoke AP discharge in the current-clamp mode (Fig. 4D). The capacity of PVINs to fire repetitively during photostimulation was examined by varying the frequency of brief photostimulation pulses (1 ms duration). APs could be reliably evoked by photostimulation at 5 Hz (99% whole cell vs 100% cell attached); however, the probability of successful AP generation decreased at higher frequencies (10 Hz: 94% whole cell vs 100% on cell; 20 Hz: 78% whole cell vs 100% cell attached; and 40 Hz: 53% whole cell vs 65% cell attached, Fig. 4E). The mean latency from photostimulation onset to AP discharge, or recruitment delay, in PVINs was 4.6 ± 0.5 milliseconds in the whole-cell recording mode and 1.3 ± 0.5 milliseconds during cell-attached recordings (Fig. 4F). These data confirm that ChR2 expression is sufficient in cells from the PV^Cre^;Ai32 cross to optically generate spiking in PVINs at frequencies of 20 Hz (in cell attached recordings) with a delay of 1.3 milliseconds after photostimulation onset.

### 3.4. Inhibitory parvalbumin-expressing interneurons provide monosynaptic inhibitory input onto neighbouring parvalbumin-expressing interneurons and other dorsal horn populations

**Figure 5.**
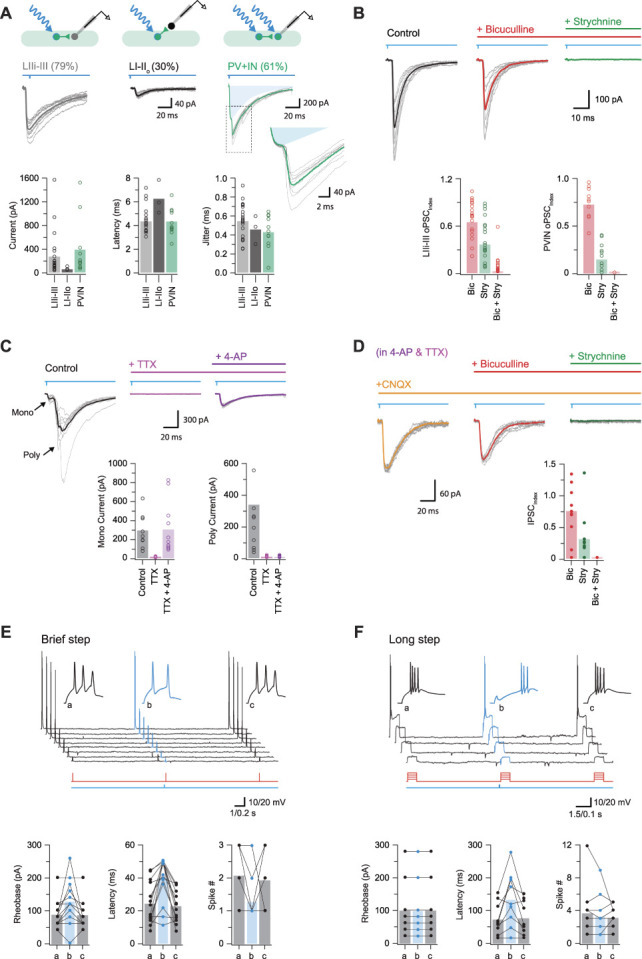
Inhibitory parvalbumin-expressing interneurons (iPVINs) provide mixed, glycine-dominant postsynaptic inhibition. (A) Upper schematics show the 3 recording configurations used to study monosynaptic connections: iPVIN to lamina IIi-III neurons, iPVIN to lamina I-IIo neurons, and iPVIN to PVIN. Voltage-clamp recordings (−70 mV) showing optically evoked inhibitory postsynaptic currents (oIPSCs) in lamina I-IIo neurons, lamina IIi-III neurons, and PVINs (10 consecutive sweeps and average overlayed). Blue shading in PVIN trace denotes underlying photocurrent isolated by a pharmacological block of synaptic events. Group data right compare oIPSC amplitude, latency, and latency standard deviation (jitter). (B) Representative oIPSC recording in CNQX (black) and after sequential bath application of bicuculline (red) and strychnine (green). Group data below summarise the effect of these drugs in lamina IIi-III neurons (left) and PVINs (right), highlighting glycine-dominant oIPSCs (ie, the IPSC index is most reduced by strychnine). (C) Representative photostimulation (black) and after sequential bath application of TTX (pink) and 4-AP (purple). Addition of TTX blocks the action potential–dependent oPSCs, which can be recovered by addition of 4-AP. Group data below summarise effects of drug application on monosynaptic currents (bottom left) and longer-latency polysynaptic currents (bottom right), highlighting the drug cocktail's ability to isolate monosynaptic responses. (D) Traces from the same recording in C (in TTX and 4-AP) after sequential addition of CNQX, bicuculline, and strychnine. Bicuculline produced a moderate reduction in the oIPSC amplitude and strychnine abolished the response. Group data below summarise this pharmacology with the oIPSC index reduced to ∼0.75 in bicuculline, ∼0.25 in strychnine, and abolished in both drugs. (E) Top traces show AP discharge responses recorded in a lamina IIi-III neuron during brief (50 ms) depolarizing step injections: (a) pretest step, (b) test step—preceded by a PVIN photostimulation, and (c) posttest step (timing of current steps in red and photostimulation in blue, below). Insets show the AP onset on expanded time. Group data plots below summarise group data showing rheobase, latency, and number of spikes in response to stimulation are all altered by preceding activation of PVINs and the associated inhibition they mediate. (F) Plot shows same general experimental approach as in (D); however, depolarizing current injections were of longer duration (500 ms). Under these conditions only spike latency was altered by preceding PVIN photostimulation, whereas rheobase and spike number were unchanged.

The postsynaptic targets of PVINs were assessed by recording from unidentified dorsal horn neurons (cells lacking YFP in laminae I-III) and PVINs (YFP-expressing cells in laminae II-III). Recordings from unidentified neurons (UNs) were further subdivided into those located within the plexus of PVIN dendrites and processes (UN laminae IIi-III) and those located dorsal to this region (UN laminae I-IIo). Given that PVINs have predominantly been studied in the context of inhibitory connections, we first used a CsCl-based internal solution to resolve inhibitory currents in the presence of CNQX to block any excitatory input. Photostimulation often elicited oIPSCs (Fig. 5A), which occurred at short latencies (4.34 ± 0.23 ms vs 6.25 ± 0.83 ms vs 4.23 ± 0.35 ms for UN:LIIi-III, UN:LI-IIo, and PVINs, respectively) and with limited jitter (0.54 ± 0.04 ms vs 0.45 ± 0.09 ms vs 0.42 ± 0.05 ms for UN:LIIi-III, UN:LI-IIo, and PVINs, respectively). Allowing for the recruitment delay of PVIN photostimulation (1.3 ms, cell attached) and conduction plus synaptic delays of ∼2 milliseconds,^50^ we conclude that these latencies and limited jitter are consistent with the existence of monosynaptic connections. Using these criteria, PVIN photostimulation resulted in monosynaptic oIPSCs in 79% (146/185) of UN:LIIi-III, 30% (16/53) of UN:LI-IIo, and 61% (67/110) of PVIN recordings in laminae II-III.

Group data comparisons (Fig. 5A) show that oIPSC amplitude was similar for both UN:LIIi-III and PVINs (both located within the PV plexus), but oIPSC amplitude was significantly smaller in recordings from UN:LI-IIo (260 ± 80 pA vs 381 ± 148 pA vs 46 ± 19 pA, respectively, *P* < 0.05). These data suggest that within laminae IIi and III, iPVINs form an extensive network of synaptic connections both with other PVINs as well as many other cells in this region. This is of particular interest given that recent genetic ablation and tetanus toxin silencing studies^8,63^ indicate these circuits play a critical role in the confinement of tactile signals to deeper layers of the dorsal horn, interpreted through specific inhibitory connections with vertical cells and cells identified by PKCγ expression.

The pharmacology of these iPVIN connections was further assessed by bath addition of the GABA_A_R antagonist bicuculline or the GlyR antagonist strychnine (Fig. 5B). For recordings from UN:LIIi-III, UN:LI-IIo, and PVINs, bicuculline application decreased oIPSC amplitude (oIPSC_index_: 0.70 ± 0.05, *P* < 0.001; 0.49 ± 0.17, *P* = 0.2; and 0.75 ± 0.05, *P* = 0.001 for UN:LIIi-III, UN:LI-IIo, and PVINs, respectively). Similarly, bath application of strychnine also reduced oIPSC amplitude, although more dramatically than bicuculline (IPSC_index_: 0.42 ± 0.06, *P* < 0.001; 0.51 ± 0.19, *P* = 0.12; and 0.17 ± 0.04, *P* < 0.001 for UN:LIIi-III, UN:LI-IIo, and PVINs, respectively). Finally, in a subset of recording that isolated either GABAergic or glycinergic oIPSCs during PVIN photostimulation, the corresponding antagonist was then added to abolish the remaining oIPSC current.

The assignment of photostimulation responses as monosynaptic was also confirmed with bath application of TTX and 4-AP (Fig. 5C), where only AP-independent “terminal release” is possible by direct ChR2-mediated depolarisation of presynaptic terminals.^17,64^ Raw photostimulation responses were first recorded (ie, without CNQX). Under these conditions, TTX alone abolished all monosynaptic responses (ie, those with short latency and low jitter), as well as any presumptive polysynaptic responses. In agreement with the criteria for monosynaptic responses, addition of 4-AP (in the presence of TTX) reinstated short-latency responses (298 ± 56 pA vs 10 ± 1 pA vs 306 ± 82 pA; baseline vs TTX vs TTX + 4-AP, respectively). CNQX was then applied to confirm isolation of iPVIN inputs, and the pharmacology of these monosynaptic oIPSCs was assessed (Fig. 5D, IPSC index: 0.76 ± 0.10 and 0.31 ± 0.10 after application of bicuculline and strychnine, respectively, n = 14). Taken together, these data confirm that both GABA and glycine are released by iPVINs with glycine being the dominant neurotransmitter in postsynaptic inhibition.

The impact of iPVIN-mediated inhibition on dorsal horn (DH) neuron excitability was assessed in current-clamp recordings from UN:LIIi-III cells, combining photostimulation with a series of brief (Fig. 5E, n = 14) or long (Fig. 5F, n = 9) depolarising current step protocols coupled with PVIN photostimulation. In brief step responses, PVIN photostimulation increased rheobase current (89 ± 11 pA vs 130 ± 17 pA, *P* = 0.012 vs 87 ± 10 pA, *P* = 0.011 for step 1, step 2, and step 3, respectively), increased the latency to AP discharge (24.0 ± 3.4 ms vs 39.5 ± 3.7 ms, *P* = 0.001 vs 22.6 ± 2.6 ms, *P* < 0.001), and reduced the number of APs in the response (2.1 ± 0.2 vs 1.3 ± 0.2, *P* = 0.003 vs 1.9 ± 0.2, *P* = 0.045). In long step depolarisations, PVIN photostimulation significantly increased the latency of AP discharge (72.9 ± 16.4 ms vs 134.3 ± 27.9, *P* = 0.013 vs 76.8 ± 17.6 ms, *P* = 0.017 for step 1, step 2, and step 3, respectively), but did not affect rheobase current (100 ± 28 pA vs 100 ± 28 pA, *P* = 1.0 vs100 ± 28 pA, *P* = 1.0) or the number of APs evoked per step (3.7. ± 1.2 vs 3.3 ± 0.9, *P* = 0.397 vs 3.1 ± 0.5, *P* = 0.746). Thus, activation of PVINs produced an overall inhibition of DH neuron discharge within the main PV plexus (laminae IIi-III).

### 3.5. Excitatory parvalbumin-expressing interneurons mediate monosynaptic glutamatergic input but rarely recruit other dorsal horn populations

**Figure 6.**
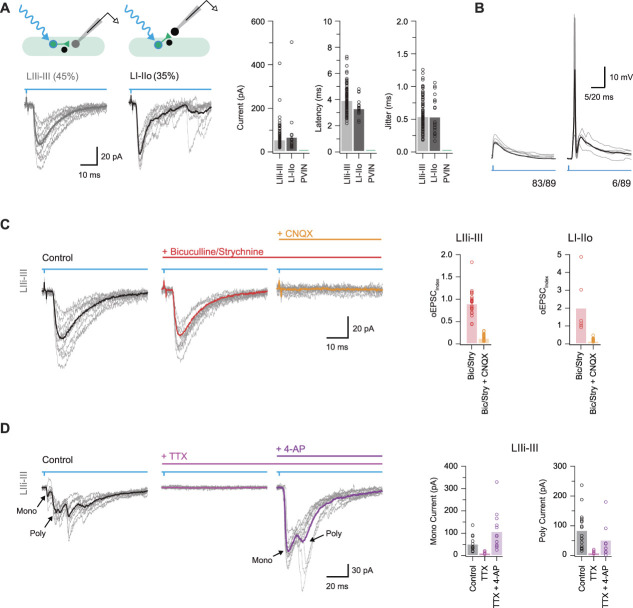
Excitatory parvalbumin-expressing interneurons are a source of monosynaptic glutamatergic excitation. (A) Upper schematics show the 2 recording configurations used to study monosynaptic excitatory connections: PVIN to lamina IIi-III neurons and PVIN to lamina I-IIo neurons. Traces below show corresponding voltage-clamp recordings (−70 mV) of optically evoked excitatory postsynaptic currents (oEPSCs, 10 consecutive sweeps and average overlayed). Note, no oEPSC responses were observed in PVIN recordings. Group data (right) compare oEPSC amplitude, latency, and latency standard deviation (jitter). (B) Current-clamp recordings (from −60 mV) showing photostimulation-evoked oEPSCs rarely induce action potential discharge in postsynaptic neurons (only 4/89 recordings featured AP discharge in oEPSP responses). (C) Representative oEPSC recording (black) with subsequent addition of bicuculline and strychnine (red) and then CNQX (orange). Block of GABA and glycine receptors had minimal effects on oEPSCs, whereas the addition of CNQX abolished the response. Group data (right) summarise the effects of this drug application regimen in lamina IIi-III (left) and lamina I-IIo (right) neuron oEPSC responses. (D) Representative oEPSCs recorded recording (black) with subsequent addition of TTX (pink) and 4-AP (purple). Group data (right) summarise the effects of this drug application regimen on monosynaptic current (left) and polysynaptic current (right). Addition of 4-AP in the presence of TTX application recovers monosynaptic oEPSCs, as well as many presumably action potential–independent polysynaptic oEPSCs. PVIN, parvalbumin-expressing interneuron.

To examine the impact of ePVIN input within the dorsal horn, we next used a potassium gluconate–based internal solution to better differentiate excitatory responses and to study action potential spiking responses in postsynaptic targets. First, photostimulation of PVINs evoked monosynaptic oEPSCs in UN:LIIi-III (45%, 103/231) and UN:LI-IIo (35%, 22/63), but not in PVINs (0%, 0/36, Fig. 6A). Importantly, oEPSCs exhibited short latency (3.91 ± 0.11 ms and 3.29 ± 0.15 ms for UN:LIIi-III and UN:LI-IIo, respectively) and limited jitter (0.53 ± 0.03 ms and 0.53 ± 0.07 ms for UN:LIIi-III and UN:LI-IIo, respectively), consistent with monosynaptic connections. Optically evoked excitatory postsynaptic current amplitude was similar in UN:LIIi-III and UN:LI-IIo cells (47.9 ± 5.6 pA and 60.5 ± 24.0 pA, respectively). The capacity of these excitatory inputs to recruit action potential discharge was assessed in current clamp in some recordings (n = 89, Fig. 6B). This showed that monosynaptic oEPSPs were rarely capable of eliciting AP discharge in postsynaptic UN:LIIi-III (4/66) or UN:LI-IIo cells (2/23), although simultaneous activation of ePVINs and iPVINs may have contributed to this observation.

To further confirm the excitatory nature of these connections, we assessed their sensitivity to bicuculline, strychnine, and CNQX application (Fig. 6C). Optically evoked excitatory postsynaptic current amplitude for UN:LIIi-III and UN:LI-IIo was not reduced by bicuculline (oEPSC_index_: 1.0 ± 0.04, *P* = 0.91 and 0.99 ± 0.09, *P* = 0.948, respectively) or strychnine (oEPSC_index_: 0.90 ± 0.09, *P* = 0.31 and 1.83 ± 0.57, *P* = 0.201, respectively) but was abolished by CNQX (oEPSC_index_: 0.12 ± 0.01, *P* < 0.001 and 0.14 ± 0.03, *P* < 0.001, respectively). Finally, TTX was applied in a subset of recordings and abolished oEPSCs in all cases. These oEPSCs could be restored, or in some cases enhanced, by the addition of 200 μM 4-AP in TTX (oEPSC_index_: 3.7 ± 1.72, *P* = 0.138; Fig. 6D), providing further evidence for the monosynaptic nature of these inputs. Consistent with the above pharmacology, the 4-AP-enhanced currents were not affected by the addition of bicuculline (oEPSC_index_: 1.03 ± 0.08, *P* = 0.732) or strychnine (oEPSC_index_: 0.97 ± 0.08, *P* = 0.786) but were abolished by CNQX (oEPSC_index_: 0.14 ± 0.05, *P* = 0.035). Taken together, these data show a subpopulation of glutamatergic PVINs provide excitatory drive to neurons in laminae IIi-III and I-IIo; however, these inputs rarely cause AP discharge in their postsynaptic targets.

### 3.6. Parvalbumin-expressing interneuron–evoked polysynaptic input arises from multiple distinct circuits

**Figure 7.**
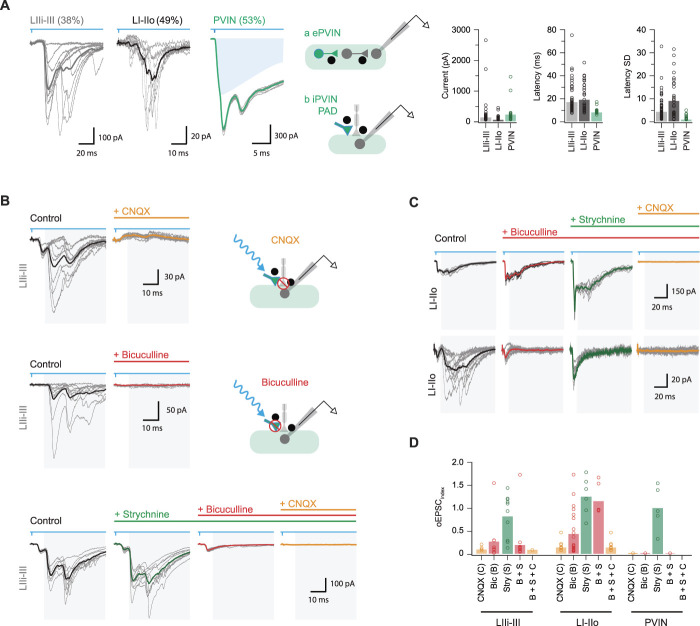
Parvalbumin-expressing interneuron (PVIN) activation evokes polysynaptic-evoked excitatory postsynaptic currents (EPSCs). (A) Traces show voltage-clamp recordings (−70 mV) of optically evoked excitatory postsynaptic currents (oEPSCs) in lamina I-IIo neurons, lamina IIi-III neurons, and PVINs (10 consecutive sweeps and average overlayed). Note these excitatory responses exhibited long latencies. Blue shading denotes underlying photocurrent in PVIN recording. Schematics (middle) summarise proposed polysynaptic PVIN circuits. Responses could arise from photostimulation of excitatory PVINs that recruit interposed excitatory INs (a, middle upper) or photostimulation may activate an inhibitory PVIN input onto a primary afferent fibre that terminates on the recorded neuron (b, middle lower). Group data (right) summarise oEPSC amplitude, latency, and latency standard deviation (jitter). These characteristics are consistent with polysynaptic oEPSCs. (B) Representative oEPSCs recorded from neurons in laminae IIi-III (black traces) and after addition of CNQX (upper left, orange trace), bicuculline (middle left, red trace), or strychnine (lower, green), bicuculline (lower, red), and then CNQX (lower, orange). Schematics (right) show the postulated circuit and site of drug action for these outcomes. GABA and AMPA receptor block abolished polysynaptic oEPSCs, whereas addition of strychnine had a minimal effect. (C) Representative oEPSCs recorded from neurons in laminae I-IIo (black traces) and after addition of bicuculline (red), strychnine (green), and CNQX (orange). Two series of traces highlight the variability in drug responsiveness of neurons in laminae I-IIo. Addition of bicuculline had a minimal effect in some recordings (upper traces), whereas bicuculline abolished polysynaptic oEPSCs in other recordings (lower traces). (D) Group data summarise the effects of drug application regimens in neurons from laminae IIi-III (left), I-IIo (middle), and PVINs (right). Note CNQX or bicuculline abolished all polysynaptic responses in PVINs and most neurons in lamina IIi-III but responses to bicuculline varied in recordings from lamina I-IIo.

In addition to short-latency (monosynaptic) oEPSCs described above, PVIN photoactivation evoked polysynaptic excitatory input (oEPSCs, Fig. 7A). These responses were resolved in UN:LIIi-III cells (38%, 87/231), UN:LI-IIo (49%, 31/63), and PVINs (50%, 18/36) and had longer latencies (16.9 ± 1.6 ms, 19.5 ± 2.2 ms, and 8.2 ± 0.6 ms for UN:LIIi-III, UN:LI-IIo, and PVINs, respectively) and exhibited greater jitter (4.3 ± 0.71 ms*,* 9.3 ± 1.6 ms, and 0.98 ± 0.27 ms) than monosynaptic oEPSCs. Polysynaptic oEPSC amplitude varied across the sample, with PVINs receiving the largest input followed by UN:LIIi-II recordings and finally UN:LI-IIo cells (235 ± 87 pA vs 140 ± 52 pA vs 68 ± 15.5 pA, respectively, *P* = 0.15), with all responses abolished by CNXQ (oEPSC_index_: 0.11 ± 0.03 *P* < 0.001; 0.16 0.04 *P* < 0.001; and 0 ± 0, *P* < 0.001; Figs. 7B–D). Current-clamp recordings showed polysynaptic oEPSCs occasionally elicited AP discharge in UN:LIIi-III (16/66) or UN:LI-IIo cells (3/23). These APs had substantially greater latency than those elicited by monosynaptic oEPSCs (25.57 ± 4.37 ms vs 6.96 ± 0.6 ms), and neurons exhibiting AP discharge received much stronger polysynaptic input than monosynaptic (593 ± 226 pA vs 140 ± 39 pA). Such responses clearly required photostimulation to recruit glutamatergic circuitry, with the ePVINs described above one obvious source of input. Despite this, we found monosynaptic oEPSCs rarely initiated AP discharge (Fig. 6B), arguing against ePVINs being a major source of polysynaptic responses.

Presynaptic inhibition arising from iPVINs is an alternative mechanism that could give rise to polysynaptic excitatory responses under our experimental conditions.^8,26^ Specifically, iPVIN photostimulation has been shown to evoke GABA-mediated primary afferent depolarisation (PAD) that is capable of eliciting neurotransmitter release from afferent terminals.^8^ This mechanism would produce longer latency polysynaptic oEPSCs as we have previously demonstrated in iPVIN photostimulation responses recorded in lamina II vertical neurons.^8^ These previous experiments showed that such connectivity is sensitive to glutamatergic, as well as GABA_A_ receptor blockade at the iPVIN-to-primary afferent synapses. Thus, we assessed the sensitivity of polysynaptic oEPSCs recorded here to bath-applied bicuculline (Figs. 7B–D). All polysynaptic oEPSCs in PVINs were abolished by bicuculline (oEPSC_index_: 0 ± 0, *P* < 0.001), which also significantly reduced polysynaptic oEPSCs in UN:LIIi-III and UN:LI-IIo cells (oEPSC_index_: 0.28 ± 0.16, *P* = 0.002 and 0.45 ± 0.11, *P* < 0.001, respectively). These data support a role for iPVIN-evoked PAD events in generating polysynaptic oEPSCs. Furthermore, given the axoaxonic nature of iPVIN input to primary afferents, it is possible that polysynaptic oEPSCs arising from these connections may persist in the presence of TTX and 4-AP. Indeed, polysynaptic oEPSCs were still detected in the presence of TTX and 4-AP in some recordings (8/18, Fig. 6D). This result supports the interpretation that the polysynaptic circuitry in question does not rely on spiking in an interposed neuron, but rather PAD in primary afferent terminals. Taken together, these findings demonstrate that iPVIN activation during photostimulation causes PAD and that this can produce polysynaptic oEPSCs by driving neurotransmitter release from primary afferent terminals under experimental conditions.

These experiments demonstrate the capacity for iPVINs to mediate GABAergic presynaptic inhibition, and we also demonstrate that glycine acts as the dominant neurotransmitter used by PVINs in postsynaptic inhibition (Figs. 5B and D). Thus, the impact of glycine receptor block on polysynaptic oEPSCs was also assessed (Figs. 7B–D). Unlike bicuculline, strychnine did not affect polysynaptic oEPSC amplitude in UN:LIIi-III, UN:LI-IIo, and PVIN recordings (EPSC_index_: 0.83 ± 0.15, *P* = 0.28; 1.26 ± 0.17, *P* = 0.192; and 1.00 ± 0.22, *P* = 1.0, respectively). This is consistent with GABA being the sole mediator of iPVIN-mediated presynaptic inhibition; however, it is also worth noting that the EPSC_index_ increased after the administration of strychnine in some UN:LIIi-III (5/10 cells tested), UN:LI-IIo (4/6 cells tested), and PVINs (2/5 cells tested) (Fig. 7D). These data suggest that glycinergic inhibition also regulates this circuitry, likely through ongoing tonic and phasic glycinergic inhibition of the photostimulated iPVINs.^30^

It is important to note that despite the above evidence, not all polysynaptic oEPSCs were blocked by bath-applied bicuculline (Fig. 7D). This observation was most prominent in LI-IIo, where the average oEPSC index was still 0.5 in bicuculline (50% reduction of response amplitude). Thus, our data also provide evidence that photostimulation of ePVINs can produce signalling through polysynaptic circuits. In fact, bicuculline and strychnine enhanced the amplitude of these responses in many cases and disinhibition even unmasked polysynaptic excitatory responses on some occasions. Together, these observations indicate that polysynaptic excitatory circuits can also be driven by ePVINs. The output of these excitatory circuits was more common in laminae I-IIo and was clearer under disinhibited conditions.

### 3.7. Disinhibition unmasks polysynaptic excitatory parvalbumin-expressing interneuron circuits and recruits postsynaptic targets

**Figure 8.**
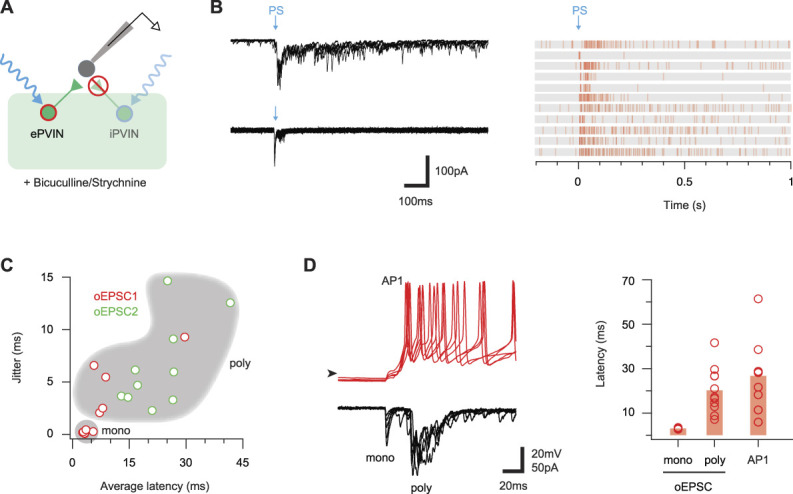
Disinhibition unmasks ePVIN-mediated polysynaptic responses. (A) Schematic summarises experimental approach, recording from Lamina I-IIo neurons during brief PVIN photostimulation (1 ms duration) in the presence of bath-applied bicuculline and strychnine. This isolated ePVIN-mediated responses and unmasked connectivity supressed by iPVIN-mediated, or ongoing, inhibition. (B) Example traces (left) show ePVIN poststimulation responses recorded in 2 lamina I-IIo neurons (upper and lower, 5 consecutive overlayed sweeps) under disinhibited conditions (bicuculline/strychnine). Responses typically contained a short-latency oEPSC, followed by varying degrees of longer-latency oEPSC activity. Raster plots (right) summarise ePVIN photostimulation responses recorded from 11 lamina I-IIo neurons. (C) Plot compares latency and jitter of the first and second photostimulation-evoked inputs (oEPSC1 and oEPSC2) from each recording summarised in (B). A cluster of short-latency (∼5 ms) and low jitter (<0.5 ms) oEPSCs exhibited monosynaptic characteristics, and a population of longer-latency, high jitter oEPSCs exhibited polysynaptic characteristics. (D) Example ePVIN photostimulation responses (left) from a lamina I-IIo neuron recorded in current-clamp (upper) and voltage-clamp (lower) modes. Note the monosynaptic and polysynaptic oEPSC responses are clear on this expanded time scale (lower), and AP discharge produced by these inputs (upper) corresponds to the polysynaptic component. Plot compares the average latency for the onset of monosynaptic oEPSCs, polysynaptic oEPSCs, and APs after ePVIN photostimulation. AP latency corresponds to the timing of polysynaptic oEPSCs in most recordings. ePVIN, excitatory parvalbumin-expressing interneuron; iPVIN, inhibitory parvalbumin-expressing interneuron; oEPSC, optically evoked excitatory postsynaptic current; PVIN, parvalbumin-expressing interneuron.

The combined observations that ePVIN inputs rarely evoked AP discharge, but that this circuitry was under ongoing inhibitory regulation, prompted additional photostimulation experiments under disinhibited conditions (Fig. 8A). These recordings were undertaken in the presence of bicuculline (10 μM) and strychnine (1 μM) to block inhibition and PAD-evoked excitation arising from iPVINs, allowing excitatory inputs and the postsynaptic excitatory networks to be unmasked. Under these conditions, brief photostimulation (1 ms pulse) produced a surprisingly extended barrage of oEPSCs, (Fig. 8B). Analysis of the latency/jitter relationship of the first and second oEPSCs in each recording (oEPSC1 and oEPSC2) showed that oEPSC1 typically exhibited monosynaptic characteristics (short latency/low jitter, Fig. 8C). In some recordings, however, oEPSC1 had polysynaptic latency/jitter characteristics, suggesting these neurons did not receive a direct ePVIN input. The second oEPSC in these responses (oEPSC2), and by extension subsequent oEPSCs, exhibited latency/jitter characteristics consistent with input through an ePVIN-activated polysynaptic circuit. Comparison of ePVIN photostimulation responses in voltage and current clamp showed that these inputs were often capable of producing AP discharge (Fig. 8D). Comparison of oEPSC1 and oEPSC2 latencies with the latency of AP discharge in corresponding recordings (3.10 ± 0.15 ms vs 20.26 ± 3.08 ms vs 26.78 ± 6.15 ms) shows that the onset of postsynaptic spiking was delayed relative to the timing of photostimulation (Fig. 8D). This relationship is compatible with postsynaptic spiking driven largely through ePVIN-mediated polysynaptic pathways, rather than monosynaptic inputs from ePVINs.

### 3.8. Postsynaptic targets of parvalbumin-expressing interneuron circuits

**Figure 9.**
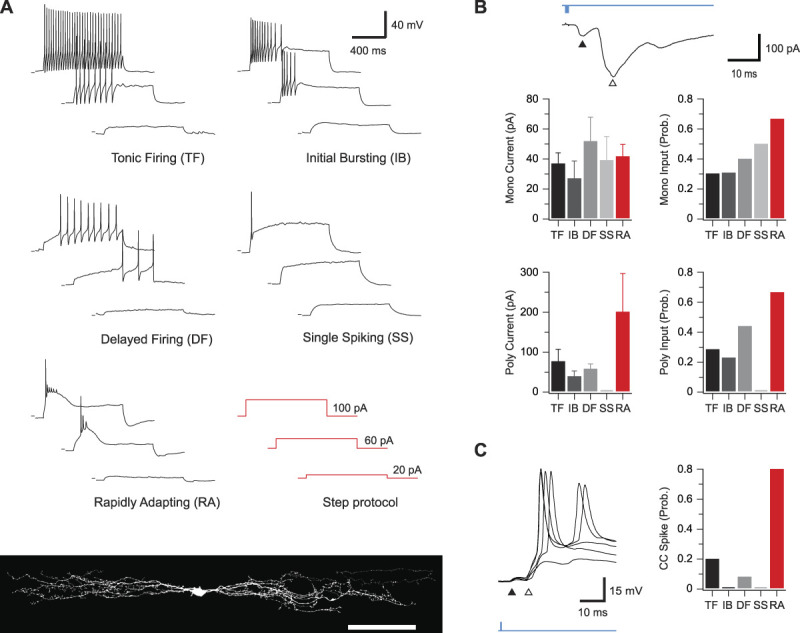
Action potential discharge responses of neurons receiving PVIN-mediated glutamatergic inputs. (A) Example traces of the 4 characteristic action potential discharge types after depolarizing current injection (bottom right): tonic firing, initial bursting, delayed firing, and single spiking, as well as another, previously unidentified, population of rapidly adapting neurons. Rapidly adapting neurons all exhibited islet cell morphology (bottom), suggesting an inhibitory phenotype. (B) Top trace is an example voltage-clamp recording from a neuron receiving monosynaptic (black arrow) as well as polysynaptic (white arrow) oEPSC input. Group data graphs (below) show the incidence of monosynaptic (top right) and polysynaptic (bottom right) oEPSCs in neurons with each type of discharge response. These data highlight the higher incidence of polysynaptic oEPSCs in the rapidly adapting population. Similarly, the amplitude of monosynaptic (top left) and polysynaptic (bottom left) oEPSCs is compared for responses recorded in each AP discharge category. Only the amplitude of polysynaptic oEPSCs differed where rapidly adapting neurons received larger amplitude inputs. (C) Trace is an example current-clamp recording from a rapidly adapting neuron receiving monosynaptic (black arrow) as well as polysynaptic (white arrow) oEPSC input. Note the polysynaptic component of the oEPSC reaches AP threshold and evokes a spike. Group data (right) highlight the increased incidence of oEPSC-evoked AP discharge in the rapidly adapting population vs all other discharge categories. oEPSC, optically evoked excitatory postsynaptic current; PVIN, parvalbumin-expressing interneuron.

Given the substantial heterogeneity described for dorsal horn neurons,^32,76^ we next assessed the AP discharge patterns of neurons receiving oEPSCs (n = 203, Fig. 9) because these features have been used to infer inhibitory or excitatory neuron phenotypes.^32,76^ Specifically, TF and IB (or adaptive firing) discharge are common in inhibitory neurons, whereas delayed firing (DF) is typical of excitatory cells. PVIN photostimulation responses were identified in neurons with a range of AP discharge profiles that included TF, IB, DF, and single spiking (SS). We also identified photostimulation responses in neurons with a distinct AP discharge pattern not commonly described. In these cells, a rapid depolarising hump was associated with burst of APs at the beginning of depolarisation (Fig. 9A). Given our sampling included recordings from lamina III, these neurons may correspond to phasic cells that exhibit pronounced spike frequency adaptation, reported in the deep dorsal horn.^69^ Morphological recovery of these neurons, herein termed rapidly adapting (RA), confirmed they exhibited extensive rostrocaudally oriented dendritic arbours as commonly observed in lamina II islet cells. Rapidly adapting neurons more often received monosynaptic oEPSCs from ePVINs than neurons with other discharge patterns (RA = 67%, 18/27; TF = 30% 19/63; IB = 31% 8/26; DF = 40%, 30/75; and SS = 50%, 6/12), although all oEPSCs were of similar amplitude (RA = 42 ± 8 pA, TF = 37 ± 7 pA, IB = 27 ± 12 pA, DF = 52 ± 16 pA, and SS = 39 ± 16 pA) (Fig. 9B). Thus, ePVINs seem to provide similar levels of input to other dorsal horn neurons with both excitatory and inhibitory characteristics.

The AP discharge properties of neurons receiving polysynaptic oEPSCs were also assessed to determine whether these connections preferentially targeted excitatory or inhibitory neurons (Fig. 9B). As for monosynaptic inputs, RA neurons more often received polysynaptic oEPSCs than neurons with other discharge patterns (RA = 67%, 18/27; TF = 29%, 18/63; IB = 23%, 6/26; DF = 44%, 33/75; and SS = 0%, 0/12). The amplitude of polysynaptic oEPSCs was also substantially larger in recordings from the RA population (RA = 201 ± 96 pA, TF = 77 ± 30 pA, IB = 40 ± 14 pA, and DF = 59 ± 13 pA), and these oEPSCs were more likely to initiate AP discharge in the RA recordings (RA = 80% 12/15, TF = 20%, 3/15; IB = 0%, 0/4; and DF = 8%, 2/25, Fig. 9C). In agreement with the postulated role of PVIN-mediated PAD in initiating these polysynaptic oEPSCs, they could be abolished by bicuculline (EPSC_index_: 0.13 ± 0.02, n = 5) or CNQX (EPSC_index_: 0.07 ± 0.01, n = 2) but were unaffected by strychnine (EPSC_index_: 1.15 ± 0.15, n = 3). Thus, RA neurons seem to receive large afferent inputs that are strongly regulated by iPVIN, whereas the afferent input regulated by this mechanism in other inhibitory populations (TF and IB) and excitatory populations (DF) is less pronounced.

### 3.9. Parvalbumin-expressing interneuron–mediated input to lamina I projection neurons

**Figure 10.**
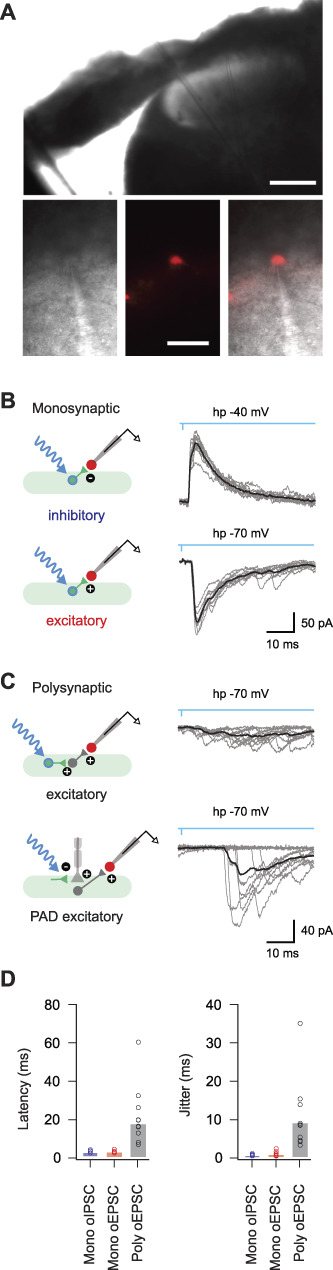
Parvalbumin-expressing interneuron (PVIN)-mediated input to lamina I projection neurons. (A) Low-magnification image (upper) shows a transverse spinal cord slice with recording pipette in place to record from a lamina I projection neuron (PN), with higher-magnification images showing the targeted recording configuration between the recording electrode and retrogradely labelled PN (brightfield left, mCherry fluorescence centre, and overlay right). (B) Photostimulation responses recorded in PNs showing characteristics of monosynaptic inhibitory (upper) and excitatory (lower) connections. Overlayed traces (gray) show 10 photostimulation (blue) trials on an expanded times scale with averaged responses (black) superimposed. Schematics (left) summarise the postulated underlying circuits between PVINs (green) and PNs (red). (C) Photostimulation responses recorded in PNs showing characteristics of polysynaptic connections. Overlayed traces (gray) show 10 photostimulation (blue) trials on an expanded times scale with averaged responses (black) superimposed. Schematics (left) summarise the postulated underlying circuits between PVINs (green) and PNs (red). Polysynaptic responses could be further differentiated into those that arose from excitatory circuits evoked by photostimulation of ePVINs (upper) and those that arose from photostimulation of iPVINs causing primary afferent depolarisation (PAD) and subsequent excitatory signalling from these terminals (lower). (D) Group data plots compare latency and jitter of photostimulation responses in monosynaptic excitatory, polysynaptic excitatory, and monosynaptic inhibitory connections. Scale bars in A (μm) = 100 upper; 40 lower. ePVIN, excitatory parvalbumin-expressing interneuron; iPVIN, inhibitory parvalbumin-expressing interneuron.

We also sought to examine PVIN-mediated input specifically to the output cells of the DH, lamina I PNs. PV^Cre^;Ai32 animals (n = 8) received bilateral parabrachial nucleus virus injections (AAV9-CB7-Cl∼mCherry) to maximally label PNs, and spinal cord slices (both transverse and sagittal planes) were subsequently prepared. Targeted recordings from mCherry-labelled PNs (Fig. 10A) used a potassium gluconate–based internal solution with oEPSCs and oIPSCs presenting as inward and outward currents, respectively (holding potentials of −70 mV and −30 mV). Under these conditions, photostimulation of PVINs evoked a range of responses (Fig. 10B). In transverse slices, monosynaptic oEPSCs exhibited bicuculline insensitivity (4/33, 12%), whereas bicuculline/strychnine-sensitive monosynaptic oIPSCs were never observed (0/28, 0%). In sagittal slices, monosynaptic bicuculline-insensitive oEPSCs (4/11; 36%) and bicuculline/strychnine-sensitive oIPSCs (2/8; 25%) were observed in PNs. These pharmacologically identified monosynaptic oEPSCs and oIPSCs exhibited short latencies (3.16 ± 0.38 ms and 3.11 ± 1.10 ms) and limited jitter (0.88 ± 0.27 ms and 0.54 ± 0.08 ms), similar to UN:LIIi-III and UN:LI-IIo cells. Thus, both the ePVINs and iPVINs provide input to PNs in lamina I.

In addition to monosynaptic inputs, some PN recordings (10/44; 22%) exhibited polysynaptic oEPSCs (Figs. 10C and D) based on a longer latency (17.81 ± 3.56 ms) and enhanced jitter (9.14 ± 1.53 ms). As above, polysynaptic oEPSCs may arise from distinct circuits involving ePVINs-mediated or iPVIN-mediated PAD, differentiated by their bicuculline sensitivity. Bicuculline reduced or abolished polysynaptic oEPSC amplitude in 3 of the 6 PNs tested and subsequent application of CNQX abolished remaining polysynaptic oEPSCs. Bicuculline sensitivity indicates a polysynaptic circuit through iPVIN-mediated PAD of primary afferents that either directly terminates on PNs or provides input through an interposed excitatory interneuron (Fig. 10C, lower). As noted above, our recent work has identified vertical cells, an excitatory interneuron population with axons arborising in lamina I, as a likely candidate to complete such a circuit.^8^ Finally, the presence of bicuculline-insensitive oEPSCs (3/6) implies that polysynaptic input to PNs is also mediated by ePVINs (Fig. 10C, upper).

### 3.10. Parvalbumin-expressing interneurons regulate nociceptive circuits

**Figure 11.**
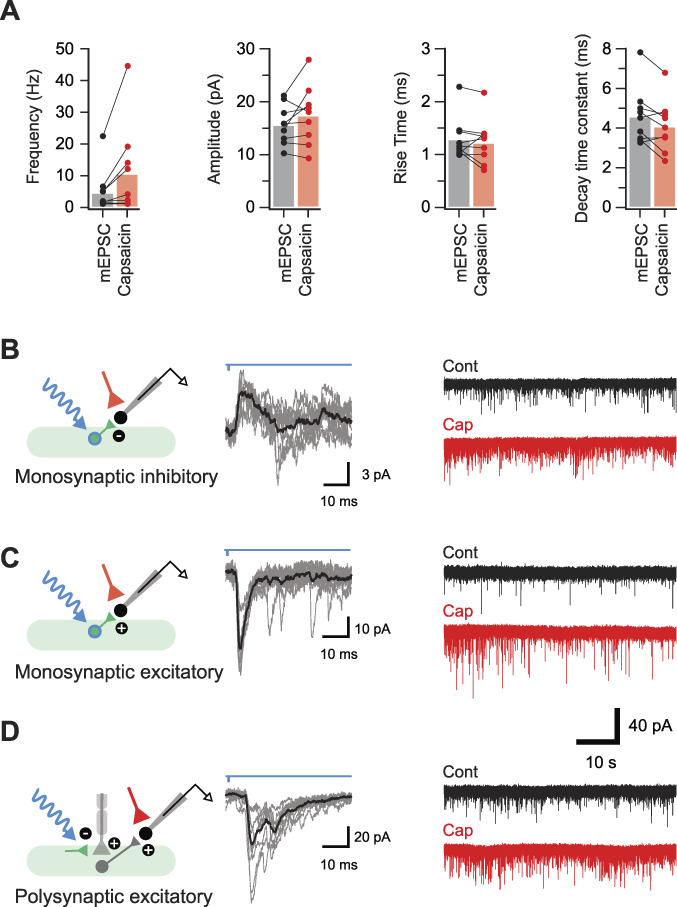
Parvalbumin-expressing interneurons modulate nociceptive circuits. (A) Plots show group data demonstrating the increase in mEPSC frequency after bath-applied capsaicin. Neurons were deemed to be capsaicin sensitive if capsaicin application increased mEPSC frequency by 3 standard deviations or more above the mean baseline rate. Miniature excitatory postsynaptic current amplitude and rise time remained unchanged, whereas decay time constant was reduced after capsaicin application. (B and C) Schematics (left) summarise the microcircuits producing photostimulation-evoked postsynaptic currents (oPSCs, 10 consecutive sweeps and average overlayed) in neurons from laminae I-IIo (middle). Continuous traces (right) show mEPSC recordings (TTX 1 μM, bicuculline 10 μM, and strychnine 1 μM) from corresponding neurons before (black) and after (red) bath application of capsaicin (2 μM). Capsaicin application increases mEPSC frequency without altering amplitude, confirming these neurons received nociceptive input. These recordings identified some neurons that received monosynaptic oIPSCs (B, short-latency outward currents during voltage clamp at −40 mV) presumably arising from photostimulation of iPVINs, neurons that received monosynaptic oEPSCs (C, short-latency inward currents during voltage clamp at −70 mV) arising from photostimulation of an ePVIN population, or neurons that received polysynaptic oEPSCs (D, longer-latency inward currents during voltage clamp at −70 mV) arising from photostimulation of iPVIN terminals that cause primary afferent depolarisation (PAD) and synaptic transmission at those terminals. ePVIN, excitatory parvalbumin-expressing interneuron; iPVIN, inhibitory parvalbumin-expressing interneuron; mEPSC, miniature excitatory postsynaptic current; oEPSC, optically evoked excitatory postsynaptic current; oIPSC, optically inhibitory postsynaptic current.

The above results confirm a significant fraction of unidentified cells in laminae I-IIo, and of PNs, received monosynaptic and polysynaptic inputs from both ePVINs and iPVINs. Given that many cells in this region receive nociceptive input, this suggests PVINs play a role in nociceptive processing as well as their established role in gating innocuous tactile inputs. To test this, a subset of recordings assessed the effect of bath-applied capsaicin on miniature excitatory postsynaptic current (mEPSC) frequency in UN:LI-IIo cells that received PVIN-mediated oEPSCs or oIPSCs (Fig. 11, n = 9). Under these conditions capsaicin causes a selective increase in mEPSC frequency only in neurons that receive direct input from TRPV1+ (nociceptive) inputs. Bath-applied capsaicin increased mEPSC frequency (4.28 ± 2.31 vs 10.33 ± 4.84 Hz, *P* = 0.049) without altering amplitude (15.4 ± 1.2 vs 17.2 ± 1.9 pA, *P* = 0.164) or time course (rise time = 1.27 ± 0.14 ms vs 1.20 ± 0.15 ms; decay time constant = 4.54 ± 0.48 ms vs 4.03 ± 0.45 ms), confirming the sample included neurons with nociceptive input (Fig. 11A). As mEPSC frequency fluctuates and the effect of capsaicin varied, a threshold was set (mEPSC frequency increase of 3 SDs above the mean baseline rate) for considering a neuron as receiving capsaicin-sensitive input. Using this criterion, two-thirds of UN:LI-IIo cells (6/9) received capsaicin-sensitive input. Assessment of PVIN photostimulation in these recording showed 1 of the 6 cells received monosynaptic iPVIN-mediated oIPSCs (Figs. 11B), 4 of the 6 cells received monosynaptic ePVIN-mediated oEPSCs (Figs. 11C), and 2 of the 6 cells received bicuculline-sensitive polysynaptic oEPSCs (Fig. 11D). Taken together, these data suggest roles for both ePVINs and iPVINs in modulating nociceptive circuits.

### 3.11. Circuit mapping parvalbumin-expressing interneuron targets in vivo

**Figure 12.**
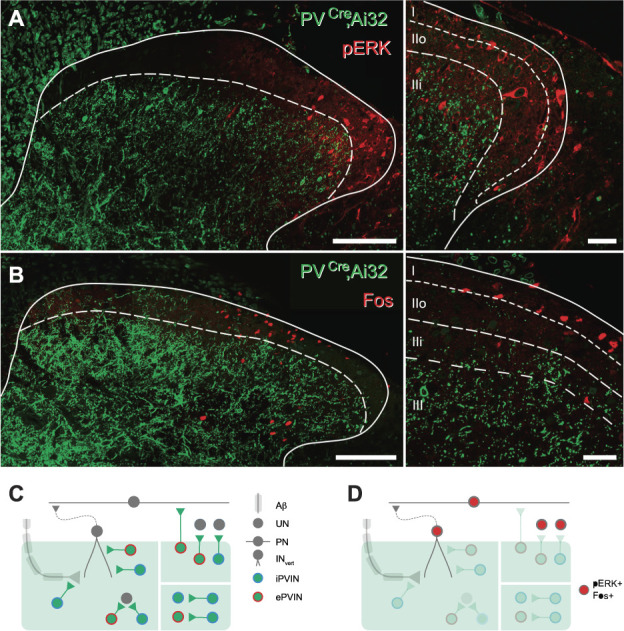
Spinal activation patterns after in vivo photostimulation of PVINs. (A) Image (left) shows maximum intensity projection of a spinal cord section taken from a PVCre; Ai32 mouse that underwent unilateral in vivo spinal photostimulation under anaesthesia. Immunolabelling for the activity marker pERK (red) and GFP to locate PVIN cells and processes (green) shows pERK expression in cells predominantly located in laminae I and IIo, with only scattered pERK-positive cells in deeper laminae. Note the absence of pERK expression in the PVIN plexus. Higher-magnification single optical section (right) from left image with lamina boundaries superimposed shows strong pERK-expressing profiles located in laminae I and IIo, implicating these cells in nociceptive processing. (B) Images show sections from an identical experiment to A, except tissue is immunolabelled for an alternative activity marker, Fos. Maximum intensity projection (left) shows Fos expression in cells predominantly located in laminae I and IIo, with only scattered Fos-positive cells in deeper laminae. Note the absence of Fos expression in the PVIN plexus. Higher-magnification single optical section (right) from left image shows strong Fos-expressing profiles located in lamina I, implicating these cells in nociceptive processing. (C) Schematic summarises identified iPVIN and ePVIN connections from channelrhodopsin-2–assisted circuit mapping experiments. Excitatory PVINs and iPVINs provide input to interneurons in LIIi-LIII including vertical cells,^8^ and iPVINs also provide presynaptic input to myelinated primary afferents (left). Inhibitory PVINs provide strong inhibition to neighbouring PVINs in LIIi-LIII (lower right). Excitatory PVINs and iPVINs provide input to interneurons and projection neurons located in LI-LIIo (upper right). (D) Schematic summarises activation of dorsal horn neurons after in vivo photostimulation of PVINs. Circuits inhibited by PVINs in LIIi-LIII and therefore unlikely to reach threshold for pERK/Fos expression are faded. By contrast, pERK/Fos expression is most pronounced in circuits in LI-LIIo, where ePVIN outputs are more likely to excite cells (including projection neurons). Scale bar (µm): A and B = 100; B = 20. ePVIN, excitatory parvalbumin-expressing interneuron; iPVIN, inhibitory parvalbumin-expressing interneuron; PVIN, parvalbumin-expressing interneuron.

To determine the pattern of neuronal recruitment after selective activation of PVINs in vivo, we assessed the pattern of activity marker labelling (pERK and Fos) in response to photostimulation of terminally anaesthetised PV^Cre^;Ai32 mice (n = 3 per group).^71^ This protocol produced reliable expression pERK and Fos in cells restricted to the spinal cord segments underlying the optic fibre (Figs. 12A and B). Most pERK-positive profiles were located in laminae I-IIo outside the PVIN plexus (449 of 676 cell counted; 161 in lamina I and 288 in lamina IIo), with fewer immunolabelled cells in the deeper laminae (161 in lamina IIi and 66 in lamina III). The general distribution of Fos-expressing cell nuclei was similar to that seen in sections immunolabelled for pERK but was not assessed formally. Surprisingly, no pERK or Fos labelling was detected in ChR2-YFP–expressing PVINs. The absence of activity markers in YFP-expressing cells was unexpected but is consistent with the extensive connectivity both between iPVINs and with ePVINs (Fig. 3) and the dominance of PVIN-mediated inhibition in this region (Figs. 12C and D). By contrast, activity marker expression in dorsal horn neurons that did not express YFP (channelrhodopsin-2) was expected and could result from glutamatergic (excitatory) inputs derived from 2 principal candidates. The most likely source would be excitatory inputs derived from axon terminals of ePVINs. Alternatively, our data show that myelinated primary afferents can produce excitatory inputs in response to iPVIN-mediated PAD (Fig. 7).^8,26^ Despite this, previous work has shown that this presynaptic inhibition–mediated signal is diminished or abolished at physiological temperatures. This leaves photostimulation of ePVINs the most likely source of both pERK and Fos induction.

## 4. Discussion

This study establishes that PV-expressing interneurons in the spinal dorsal horn comprise similar-sized excitatory and inhibitory populations. These subtypes can be defined by their morphology, and iPVINs formed homotypic and heterotypic synaptic connections with other PVINs. Excitatory PVIN-mediated excitation activated cells in laminae I and IIo, including lamina I PNs. Inhibitory PVIN-mediated inhibition was more prominent in laminae IIi and III and targeted both the central terminals myelinated afferents as well as many dorsal horn interneurons. Inhibitory PVIN-derived presynaptic inhibition was mediated by GABA, whereas postsynaptic inhibition was mediated by GABA and glycine transmission, with glycinergic signalling predominating.

### 4.1. Neurotransmitter heterogeneity of parvalbumin-expressing interneurons in the mouse dorsal horn

Our finding that half the PVINs in mouse laminae IIi-III are glutamatergic was unexpected given immunohistochemical studies in rat have reported ∼75% of PVINs in laminae I-III co-express GABA and/or glycine.^5,44^ Estimates in mouse have varied with one study reporting that ∼95% of PVINs captured in a PV^Cre^::Ai14 transgenic mouse co-express GABA and glycine.^63^ Other studies have proposed that glutamatergic PVINs are more abundant, with one study estimating 36% of PVINs were glutamatergic in PV-tdTOM BAC transgenic mice crossed with either VGLUT2^iresCre^ or VGAT^iresCre^ mice.^1^ Transcriptomic studies have also reported PV expression in both excitatory and inhibitory populations.^37^ These discrepancies may relate to the fidelity of various transgenic mouse lines but also highlight the need for careful validation of genetic labelling patterns. Our immunohistochemistry and in situ hybridisation approaches yielded broadly similar results, where ePVINs accounted for approximately half of PVINs in LIIi-III, suggesting ePVINs have been underrepresented in previous studies.

### 4.2. Excitatory and inhibitory parvalbumin-expressing interneuron connectivity

We provide the first insight into ePVINs circuits, showing this excitation is distributed throughout the dorsal horn, but oEPSCs were typically small amplitude and rarely evoked AP discharge. The inability of monosynaptic inputs to reliably drive spiking was also true under disinhibited conditions, however, released from inhibition unmasked polysynaptic pathways that produced postsynaptic spiking. This suggests that ePVINs serve an integrative role, with the ability to recruit additional excitatory populations and sum to influence dorsal horn signalling. Recent work on DH gastrin-releasing peptide (GRP) expressing excitatory interneurons has highlighted how peptide co-transmission can facilitate postsynaptic discharge.^57^ Given most ePVINs also express CCK, peptide co-transmission could similarly enhance postsynaptic responses to ePVIN inputs. Furthermore, CCK release has been implicated in tactile allodynia,^42^ CCK receptors are expressed in several dorsal horn populations,^37^ and CCK is widely distributed in mouse dorsal horn cells^36^ including CCK-expressing cells lacking PKCγ that are implicated in mechanical allodynia.^61^ Finally, the general distribution of these CCK-expressing cells overlaps with the ePVIN population, and CCK and PV are both restricted to the same transcriptomic groupings.^61^ Because recruitment of both populations activates lamina I pain circuits, it is tempting to speculate that PV expression is another defining feature of CCK cells that underlie allodynia.

A surprising difference between our Brainbow mapping and optogenetic experiments was the lack of functional excitatory inputs between ePVINs despite anatomical evidence for these connections (Fig. 3). While the reason is unclear, it is possible that ePVIN synapses represent AMPA-lacking “silent synapses” reported in the rat,^7,41,47^ although these connections are contested in adult rats.^83^ Given monosynaptic excitatory connections were recorded onto other neurons, connections between ePVINs may be rare and under sampled in our study. By contrast, direct inhibitory synaptic connections between PVINs were common, mirroring anatomical findings in the dorsal horn^38^ and other CNS regions.^74,79^ Unlike iPVINs in other regions, we found no evidence for electrical coupling or autaptic synapses.^20,53,54,58^ Nevertheless, synaptic coupling between PVINs has been shown to drive rhythmic CNS activity including gamma oscillations in the cerebral cortex^72^ and hippocampal theta/gamma synchronisation.^28,43,80^ This rhythmic activity is thought to be critical for enhanced information coding, including features of normal sensory processing and cognition.^72,77,78^ Comparable rhythmic activity has also been reported in the dorsal horn,^6,13,22,66^ with proposed roles in modality-specific coding and gating.^65^ This is consistent with iPVINs playing a “gatekeeping” role to segregate tactile and noxious signalling.^8,38,63,65^

Another striking feature of our optogenetic experiments was the widespread iPVIN connectivity to other lamina I-III neurons. Many of these cells received direct iPVIN input, consistent with previous reports of iPVIN input onto both PKCγ-expressing neurons and vertical cells.^8,63^ One other study using optogenetics to activate PVINs also found that oIPSCs were observed in most recordings.^82^ Together, these observations confirm iPVIN-mediated inhibition extends well beyond connections with PKCγ-expressing and vertical cells. Together with the ePVIN network, this widespread connectivity is directly relevant to previous optogenetic and chemogenetic studies.^8,63^ These studies reported that inactivation of PVINs caused allodynia and activation produces analgesia. The potential that these experiments manipulated ePVINs, with a likely nociceptive role, alongside iPVINs complicates these outcomes. Potential explanations include the ability for iPVIN-mediated inhibition to overwhelm ePVIN activity or the transgenic models used in this work selectively affected iPVIN function.

### 4.3. Neurotransmitter specialization at inhibitory parvalbumin-expressing interneuron inputs

Our data show iPVINs use both GABA and glycine for postsynaptic inhibition, with a clear glycinergic dominance. This matches our previous findings that PVINs themselves receive predominantly glycinergic inhibition.^30^ These conclusions are also consistent with the view that GABAergic inhibition dominates in superficial laminae, whereas glycinergic inhibition is more important in deeper laminae.^2,16,34,35,84^ Such strong glycinergic input has functional implications because glycine currents have fast kinetics with a rapid decay,^52^ features thought to be important for precisely timed inhibition in locomotor circuits.^11,26^ The need for such precise inhibitory control is equally important in the dorsal horn and also favours glycine to help segregate functionally distinct afferent signals.

Contrasting glycine-dominant postsynaptic input, we show iPVINs only use GABA for presynaptic inhibition. This agrees with a long literature demonstrating a critical role for GABA^18,23,24,29,68^ but not glycine^19,23,46^ in presynaptic inhibition. This novel difference in presynaptic and postsynaptic neurotransmitters could be explained by 2 specialised iPVIN populations or specialisation at iPVIN terminals. Well-established GABA and glycine co-expression in PVINs,^44^ the presence of GABA and glycine at most PV terminals,^75^ and the absence of glycine receptors in primary afferent terminals^55^ support the latter explanation. Together, these results add to two decades of work revising Dale's single transmitter principle for synaptic transmission.^62^ This began in the ventral spinal cord with the demonstration of GABA/glycine co-transmission,^40^ and iPVINs can now be added as a source of such signals in the dorsal horn.

### 4.4. Role of excitatory parvalbumin-expressing interneurons and inhibitory parvalbumin-expressing interneurons in pain processing

In addition to gating low-threshold inputs,^8,63^ our data suggest ePVINs and iPVINs are also capable of modulating nociceptive circuits. This finding is somewhat at odds with the dense plexus of PV axon terminals, concentrated within laminae IIi and III.^38,63^ Despite this, PVIN photostimulation evoked excitatory input in laminae I-IIo, suggesting most of these cells receive PVIN input through ventrally directed dendrites.^14,25,48,73^ Our data that lamina I PNs also receive direct PVIN input suggest a similar arrangement of dendritic extensions into the PVIN plexus. This is supported by our recent characterisation of mouse lamina I PNs, demonstrating dendritic arbours extend more ventral than previously appreciated.^9^ The observation that ePVIN inputs were small and rarely evoked AP discharge is also consistent with inputs terminating on distal dendrites and undergoing electrotonic filtering.

Our in vivo photostimulation experiments show that when fully intact and strongly activated, ePVINs can recruit signalling in lamina I, observed by pERK and Fos expression. In addition, several excitatory interneuron populations in lamina II are known to synapse with lamina I PNs including vertical cells,^15,51^ calretinin-expressing interneurons,^15,51^ and CCK-expressing interneurons.^49^ The barrage of excitatory input produced by brief ePVIN activation under disinhibited conditions supports connectivity with these excitatory networks. Given that most ePVINs co-express CCK and ePVINs provide direct input to lamina I PNs, our findings position ePVINs as an additional element of this nociceptive circuitry. Finally, the high degree of iPVIN connections suggests that ePVIN-based nociceptive circuits are under inhibitory regulation, building on the established role of iPVINs as a “gate” for innocuous stimuli.

In conclusion, this work extends our understanding of the role PVINs play in spinal sensory circuits by mapping and manipulating the activity of both excitatory and inhibitory subtypes. We establish a high degree of connectivity between iPVINs and that ePVINs make connections throughout LI-III, activating nociceptive circuitry including PNs. This implicates ePVINs in the pathological recruitment of pain circuits after loss of iPVIN inhibition. Furthermore, iPVINs not only strongly inhibit deep tactile circuits through presynaptic and postsynaptic inhibition but also contribute to the inhibition of superficial nociceptive circuitry. Together, these findings establish the diverse range of sensory modalities that require PVIN-mediated input within dorsal horn circuits.

## Conflict of interest statement

The authors have no conflicts of interest to declare.

## Appendix A. Supplemental digital content

Supplemental digital content associated with this article can be found online at http://links.lww.com/PAIN/B443.

## Supplementary Material

SUPPLEMENTARY MATERIAL
