# Supplementary material for: Diversity of inhibitory and excitatory parvalbumin interneuron circuits in the dorsal horn
Source: Pain. 2021 Jul 28;163(3):e432–52. doi: 10.1097/j.pain.0000000000002422 (PMC8832545; doi:10.1097/j.pain.0000000000002422)
Supplement: SUPPLEMENTARY MATERIAL [file jop-163-e432-s001.pdf]

Supplementary Table 1. Resources and reagents.

Reagent type (species) or resource	Designation	Source or Reference	Identifiers	Additional Information
Antibody	Goat anti-FOS	Santa Cruz Biotechnology Inc., USA	Cat# sc-52-G RRID: AB_2629503	IF (1:500)
Antibody	anti-MAPK (Erk1/2)	Cell Signalling Technology	Cat # 9106; RRID:AB_331768	IF (1:10000)
Antibody	Mouse anti-Gephyrin	SynapticSystems, Göttingen, Germany	Cat# 147021 RRID: AB_2232546	IF (1:2000)
Antibody	Chicken anti-GFP	Abcam plc., UK	Cat# ab13970 RRID: AB_300798	IF (1:500)
Antibody	Goat anti-Homer	Frontier Institute Co. Ltd, Hokkaido, Japan	Homer1-Go-Af1270	IF (1:1000)
Antibody	Chicken anti-mCherry	Abcam,Cambridge, UK	Cat# Ab205402 RRID: AB_2722769	IF (1:5000)
Antibody	Rat anti-mTFP	Kerafast Inc., Boston, MA, USA	Cat# EMU103	IF (1:500)
Antibody	Goat anti-parvalbumin	SWANT, Belinzona, Switzerland	Cat# PVG-214 RRID: AB_2313848	IF (1:500)
Antibody	Guinea pig anti-parvalbumin	Frontier Institute Co. Ltd, Hokkaido, Japan	Cat # PV-GP-Af1000 RRID: AB_2336938	IF (1:500)
Antibody	Rabbit anti-Pax2	Invitrogen; Thermo Fisher Scientific, UK	Cat# 71-6000 RRID: AB_2533990	IF (1:1000)
Antibody	Rabbit anti-Pax2	Sigma-Aldrich, St. Louis, MO, USA	Cat# HPA047704 RRID: AB_2636861	IF (1:200)
Antibody	Guinea pig anti-TagRFP	Kerafast Inc., Boston, MA, USA	Cat# EMU107	IF (1:500)
RNA probe	GAD1	ACD BioTechne; Newark, CA https://acdbio.com	Cat# 400951	
RNA probe	Slc17a6	ACD BioTechne; Newark, CA https://acdbio.com	Cat# 319171	
RNA probe	PValb	ACD BioTechne; Newark, CA https://acdbio.com	Cat# 421931	
RNA probe	CCK	ACD BioTechne; Newark, CA https://acdbio.com	Cat# 402271	
Genetic reagent (*M. musculus*)	PV^{Cre} : B6;129P2-$Pvalb^{tm1(cre)Arbr}$/J	The Jackson Laboratories, USA	Cat# JAX:08069 RRID: IMSR_JAX:008069	
Genetic reagent (*M. musculus*)	Ai9: B6.Cg-Gt(ROSA)$26Sor^{tm9(CAG-tdTomato)Hze}$/J	The Jackson Laboratories, USA	Cat# JAX:007909 RRID: IMSR_JAX:007909	

Genetic reagent (*M. musculus*)	Ai32: B6;129S-Gt(ROSA)26Sor$^{tm32(CAG-COP4*H134R/EYFP)Hze}$/J	The Jackson Laboratories, USA	Cat# JAX:012569 RRID: IMSR_JAX:012569	
Genetic reagent (*AAV virus*)	AAV9-EF1a-BbTagBY (AAV-BB1)	addgene, USA	Catalog # 45185-AAV9	see Cai et al., 2013
Genetic reagent (*M. musculus*)	AAV-EF1a-BbChT (AAV-BB2)	addgene, USA	Catalog # 45186-AAV9	see Cai et al., 2013
Genetic reagent (*M. musculus*)	AAV9-CB7~Cl-mCherry	addgene, USA	Catalog# 105544-AAV9	
Software algorithm	Neurolucida for Confocal Software	MBF Bioscience, VT, USA	https://www.mbfbioscience.com/neurolucida	
Software algorithm	Neurolucida explorer	MBF Bioscience, VT, USA	https://www.mbfbioscience.com/neurolucidaexplorer	
Software algorithm	Zen Black	Carl Zeiss, Germany	https://www.zeiss.com/microscopy/int/products/microscope-software/zen.html	
Software algorithm	Orange v3.2	Demšar et al., 2013		
Software algorithm	Qupath	Bankhead et al., 2017		
Software algorithm	TeraPlot	Kylebank Software Ltd., Ayr, UK		

Figure S1 (Relates to Figures 2 and 3). Fidelity of tdTOM labelling in different CNS regions in the PV^{Cre};Ai9 mouse line. The colocalisation of tdTom-expression and immunolabelling for PV was assessed in several different regions of the CNS. **A**, The general distribution of tdTOM cells in the lumbar spinal cord matched the general distribution of PV-IR cells, with a dense plexus of cells in laminae IIi and III, numerous cells in lamina V, and more scattered cells in ventral horn laminae VII and VIII. **B**, Most tdTOM cells in laminae IIi and III (asterisk) co-expressed PV-IR, although several PV-IR cells showed no labelling for tdTOM (arrows). **C-F**, The fidelity of tdTOM expression in PV-IR cells was much higher in the ventral horn (**C**), CA1 of the hippocampus (**D**), the dentate gyrus (**E**) and the cerebellum (**F**) with high incidence of co-expression of tdTOM labelling (red) in PV-IR cells (green), and PV-IR in tdTOM cells. Abbreviations panel D: S.O. = *stratum oriens*, S.P. = *stratum pyramidale*, S.R. = *stratum radiatum*. Abbreviations panel E: Mol = molecular layer, GCL =granule cell layer, Pol = polymorphic layer. Abbreviations panel F: Mol = molecular layer, PCL = Purkinje cell layer, GL = granular cell layer. Scale bars (μm): A = 250; B = 20; C = 50; D-F = 100.

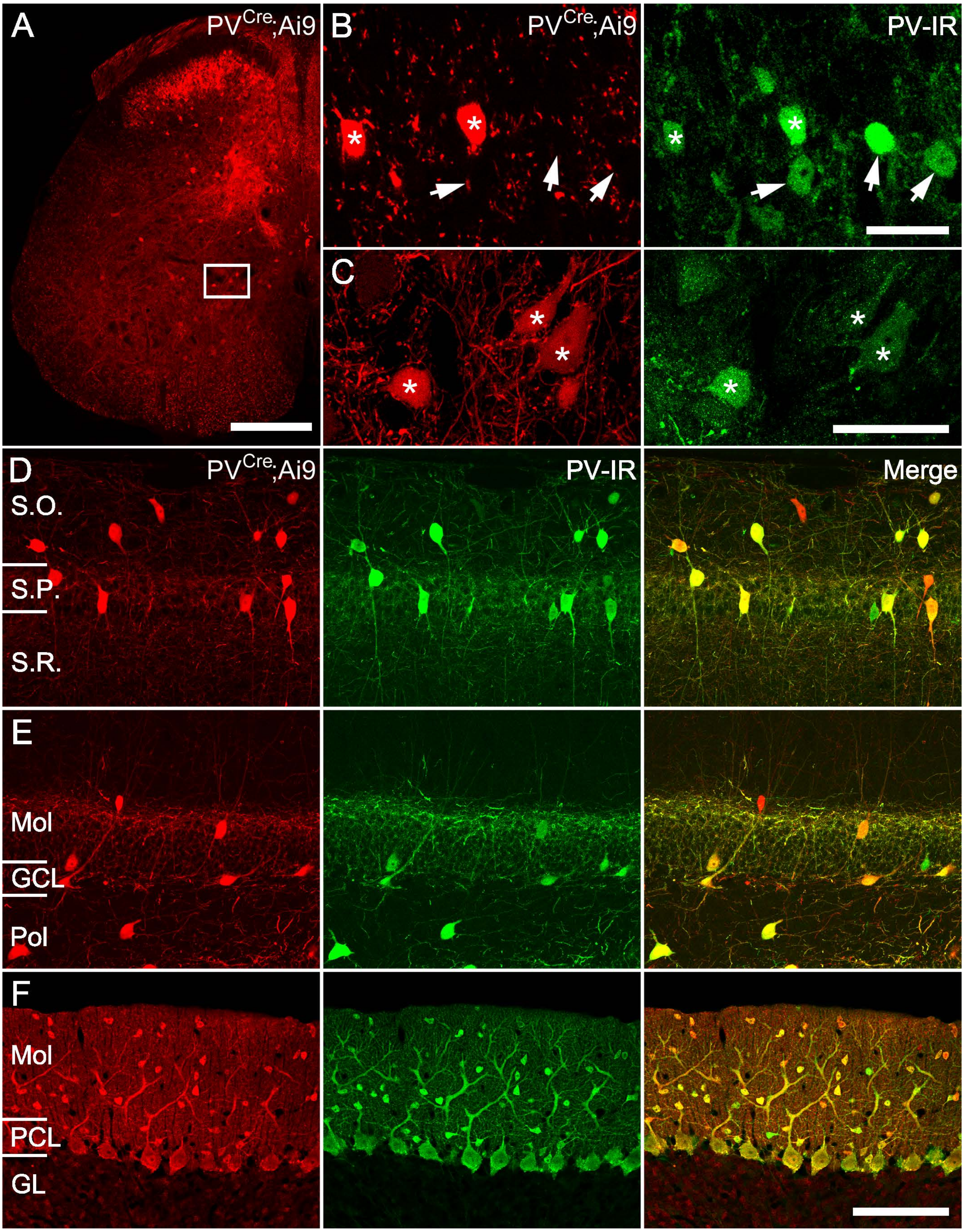
A
PVCre;Ai9
B
PVCre;Ai9
PV-IR
C
D
PVCre;Ai9
S.O.
S.P.
S.R.
PV-IR
Merge
E
Mol
GCL
Pol
F
Mol
PCL
GL
